# Intestinal Mucin Glycosylation: Structural Regulation, Homeostasis Maintenance and Disease Association

**DOI:** 10.3390/biom15111552

**Published:** 2025-11-05

**Authors:** Yunye Li, Jia Pan, Huimin Liu, Chuanguo Liu

**Affiliations:** 1 Department of Physiology, School of Medicine, Shandong University of Traditional Chinese Medicine, No. 4655, Daxue Road, Changqing District, Jinan 250355, China; 2 Experimental Center, Shandong University of Traditional Chinese Medicine, No. 4655, Daxue Road, Changqing District, Jinan 250355, China

**Keywords:** mucin glycosylation, intestinal barrier, gut microbiota, inflammatory bowel disease (IBD), intestinal homeostasis

## Abstract

The intestinal barrier is a complex configuration that defends against external assaults and maintains intestinal health. Disruption of barrier function can lead to intestinal inflammation and various diseases. Mucins are the primary structural components of the intestinal barrier, and their extensive glycosylation is critical for their protective function. Mucin glycans enhance the physicochemical integrity of the mucus barrier, protect against enzymatic degradation, modulate host immune responses, and shape the gut microbiota by providing adhesion sites and selective nutrient sources. While proper glycosylation maintains barrier integrity, supports a balanced microbial ecosystem, and limits unnecessary immune activation, its disruption leads to compromised barrier function, microbial dysbiosis, increased intestinal permeability, and ultimately contributes to the development of chronic colitis and colorectal cancer. Therefore, mucin glycosylation plays a crucial role in preserving intestinal barrier integrity and preventing colonic diseases. This review summarizes the classifications and structural features of intestinal mucin glycosylation, elucidates their roles in maintaining barrier function and their pathological alterations in intestinal disorders, and highlights the implications of mucin glycosylation for precision diagnosis and targeted therapy of intestinal diseases.

## 1. Introduction

The intestinal barrier is a multilayered system that includes a physical barrier formed by tightly joined epithelial cells and an overlying mucin-rich mucus layer, an immune barrier composed of mucosal immune cells and secretory IgA, and a microbial barrier established by commensal microbiota that colonize the intestinal lumen [1,2]. This hierarchical structure is essential for maintaining internal homeostasis by separating the dense gut microbiota from the host, thereby preventing inappropriate immune activation and infection. Those relevant components operate in dynamic balance to preserve mucosal integrity and homeostasis [2,3]. Dysfunction of this barrier allows bacteria and their products to cross the mucosa, leading to excessive inflammation and tissue damage. This disorder contributes to the development of various intestinal disorders, including inflammatory bowel disease, metabolic syndrome, and certain autoimmune conditions [3,4].

Mucins, key components of the intestinal barrier, are abundantly expressed on epithelial surfaces and in secretions. They not only constitute the structural foundation of the physical barrier but also actively participate in immune regulation and host–microbiota interactions [5,6]. Based on their structural and functional characteristics, mucins are broadly classified into secreted and membrane-bound types [7]. Secreted mucins, such as MUC2 produced by goblet cells, form dense, gel-like mucus layers that cover the intestinal epithelium, which prevent direct contact between microbes or toxins and epithelial cells while providing niches and nutrients for commensal bacteria. The extensive O-glycosylation of secreted mucins endows the mucus gel with viscoelasticity and resistance to enzymatic degradation, while creating diverse glycan motifs that mediate microbial adhesion, nutrient exchange, and immune tolerance at the mucosal surface [8,9]. Membrane-bound mucins (e.g., MUC1, MUC3, MUC12, and MUC13) are primarily expressed on epithelial surfaces. The highly glycosylated extracellular domains form a glycocalyx barrier that enhances epithelial resistance to pathogen adhesion and invasion and modulates cellular signaling and local immune responses [10]. Mucin glycosylation is a complex enzymatic process catalyzed by glycosyltransferases in the Golgi apparatus [11]. It begins with the addition of N-acetylgalactosamine (GalNAc) to serine or threonine residues on mucin core proteins, followed by sequential extension into diverse O-glycan chains containing galactose, fucose, N-acetylglucosamine, and sialic acid [12,13]. This process results in considerable heterogeneity and structural diversity. Extensive glycosylation is essential for the biological functions of mucins, which enhances hydration and gel-forming properties, increases resistance to microbial enzymatic degradation, and provides receptors or nutrients for selective microbial colonization, thereby protecting against pathogen invasion [6,14]. Abnormal mucin glycosylation impairs the mucus barrier and is commonly observed in intestinal infections and inflammatory conditions. In diseases such as active ulcerative colitis, the mucus layer becomes thinner and mucin glycosylation is disrupted. These changes are accompanied by microbial dysbiosis and increased intestinal permeability. Together, these changes contribute to chronic inflammation and colorectal tumorigenesis [14,15]. Thus, mucin glycosylation is essential for maintaining mucus barrier stability. Accordingly, this review examines how the structure and regulation of the diverse forms of intestinal mucin glycosylation coordinate the physical, immune, and microbial barriers to maintain homeostasis; how disruptions in these processes contribute to major intestinal diseases; and how these insights inform glycan-based diagnostics and targeted therapies. The following sections discuss the structure of mucin glycans, their biosynthetic pathways, roles in barrier function, aberrant glycosylation patterns in colonic diseases, and emerging glycosylation-targeted therapeutic strategies.

## 2. Types and Biological Characteristics of Intestinal Mucin Glycosylation

**Figure 1:**
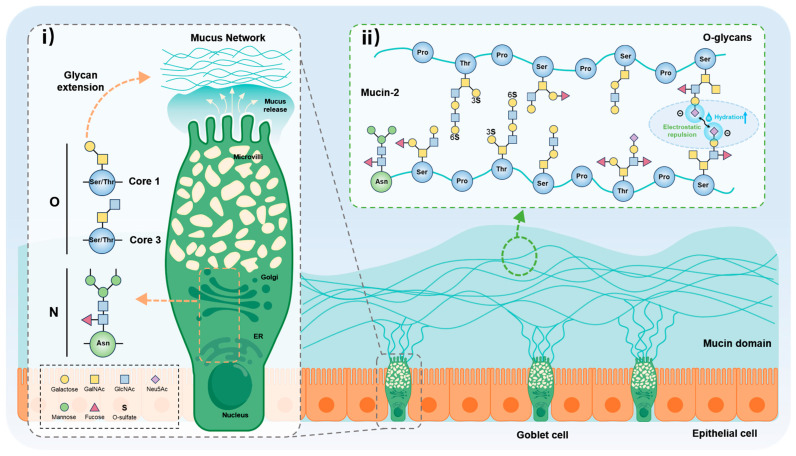
Major Glycosylation Types of Intestinal Mucins. (**i**) Intestinal mucins secreted by epithelial cells display highly complex glycan modifications, broadly categorized into core and terminal glycosylation. Core glycosylation, comprising primarily O-glycans and a smaller proportion of N-glycans, serves as the structural backbone. O-glycans (such as core 1 and core 3 structures) impart high hydrophilicity to mucins, maintaining the gel-like structure and resistance to enzymatic degradation of the mucus layer, whereas the limited N-glycans are critical for proper mucin folding and secretion. (**ii**) Terminal sialylation and sulfation confer strong negative charges, enhancing mucus hydration and resistance to microbial proteolysis. Fucosylation promotes homeostasis by facilitating interactions with commensal bacteria, although pathogens may also exploit this modification.

Intestinal mucins secreted by mucosal epithelial cells exhibit highly complex glycosylation, which can be broadly categorized into core glycosylation and terminal modifications. Core glycosylation involves the initial assembly of O-linked glycans, along with minor additions of N-linked glycans, onto the mucin peptide backbone. These core structures serve as scaffolds for subsequent terminal modifications [12,16]. Terminal glycosylation refers to the addition of specific monosaccharides such as sialic acids, sulfate groups, or fucose to core oligosaccharide structures, resulting in terminal glycans with distinct biological functions [12,17,18]. Each type of glycosylation follows a distinct biosynthetic pathway and regulatory mechanism, which collectively influence mucin conformation, physicochemical properties, and biological activity [19] (Figure 1).

### 2.1. O-Glycosylation Modification

Mucin-type O-glycosylation is one of the most common and structurally diverse post-translational modifications in eukaryotes. It is particularly abundant in mucosal secretory proteins and mediates various physiological functions [20]. O-glycosylation is initiated in the Golgi lumen, where polypeptide N-acetylgalactosaminyltransferases (GALNTs) transfer an N-acetylgalactosamine (GalNAc) residue to serine or threonine residues on target proteins, forming the initial GalNAc-Ser/Thr structure known as the Tn antigen [9]. Tn antigens are typically extended to generate up to eight distinct core oligosaccharide structures (cores 1–8), among which cores 1–4 are the most prevalent in human mucins [21,22]. After core formation, mucin O-glycans undergo terminal modifications such as sialylation, fucosylation, and sulfation, resulting in specific antigenic epitopes (e.g., T antigen, Lewis antigens, sulfated glycans). In colonic tissues, the composition of O-glycans is determined by the expression of glycosyltransferases, which is regulated by cell type and microenvironmental factors [23]. Goblet cells express high levels of core 3 synthase β1,3-N-acetylglucosaminyltransferase (B3GNT6), which extends GalNAc-Ser/Thr to form core 3 O-glycans [24]. In contrast, neighboring absorptive cells primarily utilize the core 1 pathway, mediated by core 1 β1,3-galactosyltransferase (C1GALT1) and its chaperone Cosmc [23]. Local microenvironmental factors such as cellular signaling molecules [25], microbial metabolites [26], and hypoxia [27] dynamically regulate glycosyltransferase gene expression and influence O-glycan biosynthetic pathways [28]. A concise, enzyme-level summary of mucin-type O-glycan biosynthesis and inflammation/microbiome-responsive regulation is provided in Table 1 below. Elevated Golgi luminal pH mislocalizes glycosyltransferases and remodels O-glycan termini; in intestinal epithelium, Golgi oxygen sensing also regulates mucin terminal glycosylation, changes that correlate with mucus hydration and charge [29,30].

Beyond basic biosynthesis, intestinal mucin O-glycosylation has experimentally supported roles in shaping mucin conformation, barrier integrity, signaling interfaces, and immune regulation in the gut [22]. In secreted MUC2, dense clustering of O-glycans generates extended “bottle-brush” PTS domains that sterically shield the peptide backbone and underlie the hydrated gel architecture of the inner mucus layer [31]. Loss of epithelial core-1 O-glycans breaches the inner colonic mucus, allows bacterial approach to the epithelium, and elicits spontaneous colitis in mice [32]. Complementarily, core-1 and core-3 O-glycans together protect mucins from bacterial proteases and maintain the integrity of the colonic inner mucus layer in vivo [33]. At the microbe–host interface, defined O-glycan epitopes on colonic mucins govern adhesion by the mucin forager Akkermansia muciniphila, directly linking the mucin O-glycome to commensal colonization dynamics [34]. Consistently, glycan-foraging strategies in mucin-degrading bacteria demonstrate how O-glycan codes shape mucosal ecology and influence host physiology [35]. Clinically and experimentally, truncation to Tn/sialyl-Tn on intestinal mucins is associated with heightened mucosal inflammation and neoplastic progression, including in long-standing ulcerative colitis [36]. When the mucus barrier is compromised, bacteria penetrate the normally impenetrable inner layer and reach the epithelium, underscoring the functional importance of structurally intact, O-glycan-rich MUC2 [37].

O-glycans constitute ~50–80% of intestinal mucin mass by compositional and glycoproteomic analyses, explaining the bottlebrush conformation and high water content [38]. They impart a rigid, bottlebrush-like conformation that forms the principal framework of the intestinal mucus barrier [39]. Extensive O-glycosylation endows mucins with high hydrophilicity and viscosity, enabling secreted mucus to form a dense gel layer that physically blocks microbial invasion and increases resistance to enzymatic degradation [10]. Defined mucin O-glycan epitopes (e.g., Lewis antigens, terminal sialylation/fucosylation) mediate adhesion and provide carbon sources for mucin-degrading taxa [32]. The complex glycan chains in the outer mucus layer provide carbon sources for symbiotic bacteria, many of which express glycosidases that metabolize these glycans without disrupting the integrity of the mucus barrier [7]. Genetic disruption of core-1/core-3 O-glycan synthesis results in inner-layer thinning and bacterial penetration with increased susceptibility to colitis and tumorigenesis, providing causal evidence for O-glycans in inner-layer impermeability [3,40]. Impaired O-glycan synthesis, such as the loss of key glycosyltransferases, weakens the mucus layer and increases its susceptibility to bacterial penetration [41]. In murine models, knockout of the core 3 O-glycan–synthesizing enzyme B3GNT6 leads to a marked reduction in Muc2 mucin in the colonic mucosa, increased intestinal permeability, and greater susceptibility to colitis and colorectal tumorigenesis following pathogenic challenge or chemical injury [36,42]. Failure of terminal extension yields Tn/sTn accumulation that associates with active inflammation and increased neoplastic risk; in mice, core-3 deficiency (B3gnt6^−/−^) causes inner-layer thinning, bacterial penetration, and heightened susceptibility to colitis and cancer, indicating causal risk of truncated terminals [40]. This is evidenced by the accumulation of truncated, aberrant antigens (e.g., Tn and sTn) in inflammatory and tumor settings, highlighting the pathological significance of defective glycan extension [33]. Therefore, intact O-glycosylation is essential for maintaining mucin structural integrity and barrier function, whereas its dysregulation impairs mucosal defense and contributes to disease pathogenesis.

**Table 1:** Key mucin-type O-/N-glycan structures, transferases, and microbiome-/inflammation-responsive regulation.

Mucin Glycan Structure (Intestinal)	Principal Transferase(s)	Microbiome-Regulated Expression in Inflammatory Settings	References
O-GalNAc initiation (Ser/Thr)	GALNT1-20	Family establishes site-selective initiation; inflammation-linked shifts in epithelial differentiation can modulate GALNT usage, but direct microbiome-driven regulation in IECs remains limited.	[43]
Core-1 (T antigen)	C1GALT1 with C1GALT1C1/COSMC	Tumor-associated COSMC dysfunction yields Tn/sTn truncation; direct microbiome regulation is not established, while inflammatory neoplasia provides selective pressure.	[44]
Core-2 branching	GCNT1	Supports Lewis/sialyl-Lewis elaboration relevant to leukocyte and microbial lectins; inflammation can bias extension, although microbiome-direct induction in IECs remains to be defined.	[45]
Core-3	B3GNT6	Frequently downregulated in CRC with adverse outcomes; signals along colitis–cancer continuum suggest inflammation-linked remodeling, direct microbiome control not proven.	[46]
Core-4 (from core-3)	GCNT3	Intestinal expression builds dense, protease-resistant scaffolds; regulation in active inflammation requires further definition.	[47]
Terminal α2,6-sialylation on O-GalNAc (sTn)	ST6GALNAC1	Macrophage–epithelium crosstalk in UC/CACC upregulates ST6GALNAC1, producing MUC1-sTn under chronic inflammation.	[48]
Terminal α2,3-sialylation on core-1	ST3GAL1/2/4	Infection/inflammation increases ST3GAL1 and epithelial sialylation in vivo, indicating context-responsive control relevant to mucin termini.	[49]
Terminal α1,2-fucosylation	FUT2	IL-22/STAT3 induces Fut2 in IECs during injury/repair, increasing α1,2-fucosylation and modulating commensal niches (microbiome-responsive via IL-22 axis).	[50]
Lactosamine extension (poly-LacNAc)	B3GNT7	IL-22 upregulates B3GNT7 in human IECs, coupling damage cues to extended backbones that scaffold terminal fucosylation.	[51]
Sulfation of mucins	CHST family/PAPSS2	Active UC shows reduced sulfation of mucins in human colonic tissue, indicating inflammation-linked loss; direct microbiome control is not yet defined.	[52]
N-glycan transfer (ER)	OST (STT3A/B)	Ensures en bloc transfer for secretory cargo; ER stress in goblet cells during inflammation compromises mucin biogenesis.	[53]
N-glycan branching (Golgi)	MGAT1/2/5	Branching supports folding/trafficking; epithelial stress in colitis associates with misfolded MUC2 and barrier failure.	[54]
N-glycan α2,6-sialylation	ST6GAL1	Epithelial stress programs tune ST6GAL1 in secretory pathways; context-responsive in inflammation.	[42]
N-glycan core fucosylation	FUT8	Modulates lectin binding and receptor trafficking; inflammation-responsive regulation in IECs requires further definition.	[42]

### 2.2. N-Glycosylation Modification

In addition to abundant O-glycans, intestinal mucins carry a limited number of N-linked glycans that are important for maintaining structural and functional integrity. In the human colon, MUC2 carries a limited set of N-glycans identifiable by enzymology and MS; these N-glycans cooperate with ER chaperones to promote dimerization and early assembly, coupling to subsequent Golgi O-glycosylation and granule maturation [55,56]. During N-glycosylation, oligosaccharyltransferase co-translationally transfers a preassembled 14-sugar precursor (Dolichol-PP-GlcNAc_2_Man_9_Glc_3_) to the Asn-X-Ser/Thr motif of MUC2. This precursor is then precisely trimmed and extended into mature high-mannose, hybrid, or complex N-glycan structures by glycosidases and glycosyltransferases in the endoplasmic reticulum and Golgi apparatus [57]. Key N-glycan enzymes and their inflammation-linked regulation are summarized in Table 1 above. N-glycosylation proceeds via the OST–ER trimming–Golgi extension cascade; tunicamycin blockade of N-glycan transfer slows MUC2 dimer/assembly and impairs secretion, and misfolded Muc2 in Winnie/Eeyore models links folding defects to spontaneous colitis [18,58].

During MUC2 biosynthesis in goblet cells, co-translational N-glycosylation in the rough endoplasmic reticulum (ER) enables calnexin/calreticulin lectin-chaperone engagement to assist folding and early oligomerization of the nascent mucin polypeptide [59]. Classical pulse-chase studies showed that inhibition of N-glycosylation with tunicamycin slows MUC2 dimerization and perturbs higher-order assembly in the ER, demonstrating a causal requirement for N-glycans at this step [56]. Authoritative mucin biology reviews further document that MUC2 forms disulfide-stabilized dimers in the ER and then traffics to the Golgi for extensive O-glycosylation prior to packaging and secretion [12]. Contemporary summaries of mucus biogenesis concur that ER dimerization precedes Golgi O-glycosylation and granule maturation, linking N-glycan-assisted folding to downstream assembly and export [60]. In vivo, models of Muc2 misfolding and ER stress (e.g., Winnie/Eeyore) demonstrate reduced stored mucin, a compromised mucus barrier, and spontaneous intestinal inflammation [54]. Moreover, modulation of inflammatory signaling in this model alters ER stress and mucus output, linking N-glycan-assisted folding with downstream barrier competence [61]. Mechanistic overviews of mucin assembly further establish that disulfide-bonded C-terminal dimerization occurs in the ER, followed by extensive O-glycosylation in the Golgi prior to packaging and secretion [62]. Extending to intestinal membrane mucins, mucin-centric reviews indicate that N-glycosylation supports proper folding and polarized delivery of heavily O-glycosylated ectodomains that contribute to the epithelial glycocalyx [63]. Recent genetic and mechanistic work directly connects intact mucin N-glycosylation to maturation, downstream glycosylation, mucus layering, and protection from dysbiosis-associated inflammation [64].

MUC2 N-glycosylation promotes binding to the calnexin/calreticulin chaperone system and supports conformational maturation. Inhibition of N-glycan transfer by tunicamycin delays MUC2 dimerization and higher-order assembly, demonstrating a causal role in early folding [33,65]. For instance, an N-glycan located at the ninth asparagine in the C-terminal region of MUC2 slows its folding, allowing newly synthesized monomers sufficient time to form stable dimers [56]. Removal of N-glycans impairs MUC2 dimer and polymer formation. In addition to promoting multimeric assembly, N-glycans enhance mucin stability by preventing premature degradation and regulating mucin localization and secretion at the cell surface [35,66]. Furthermore, cancerous tissues often exhibit elevated levels of highly branched N-glycans, which enhance cell adhesion and migration, thereby promoting tumor invasion and metastasis in cancerous epithelial cells [37,67]. In conclusion, N-glycosylation contributes to mucosal barrier integrity by ensuring proper mucin folding, stability, and secretion, whereas aberrant N-glycan alterations can compromise barrier function and facilitate disease progression.

### 2.3. Sialylation Modification

Sialylation is the enzymatic addition of sialic acids—typically N-acetylneuraminic acid (Neu5Ac)—to the termini of glycans and is one of the final steps in mucin glycosylation. In both O- and N-linked mucin glycans, sialic acids are typically attached to terminal galactose residues via α2,3 or α2,6 linkages, catalyzed by specific sialyltransferases. ST3Gal enzymes mediate α2,3-sialylation of galactose [68], ST6Gal enzymes catalyze α2,6-sialylation of galactose [69], and the ST6GALNAC family transfers sialic acid to the α2,6 position of GalNAc residues, such as converting Tn antigens to sTn antigens [70]. In intestinal epithelial cells, various sialyltransferases act cooperatively to generate dense sialic acid capping on mucin O- and N-glycans. For example, human colonic goblet cells express high levels of ST6GALNAC1, the primary α2,6-sialyltransferase for mucin O-glycans. Its expression is upregulated by microbe-associated molecular patterns (e.g., LPS), thereby enhancing terminal sialylation [70,71]. Pathological stimuli, such as inflammatory cytokines or hypoxia, can alter sialyltransferase expression. For instance, hypoxia induces ST3Gal-I expression in certain tumor cells, leading to increased levels of sialylated Lewis antigens (e.g., sialyl-Lewis^x) [8]. Overall, sialylation is modulated by both genetic and environmental factors. Its tight regulation is essential for maintaining the mucus barrier function.

The terminal sialic acids on mucins confer high hydrophilicity and a strong negative charge to the mucus layer, supporting its gel-like structure and barrier integrity. Electrostatic repulsion between negatively charged glycans promotes the formation of an expanded, porous network that restricts bacterial access to the epithelial surface [35]. Insufficient sialylation compromises this barrier. Mice lacking functional ST6GALNAC1 have colonic mucus that is more susceptible to bacterial enzymatic degradation, accompanied by dysbiosis and spontaneous intestinal inflammation resembling early-onset inflammatory bowel disease [35]. Mechanistically, dominant mucin degraders first remove terminal sialic acid and fucose via sialidases/fucosidases before accessing core O-glycans; genetic and biochemical mapping shows that these terminal caps slow mucin utilization and niche expansion [41]. Conversely, excessive sialylation can have deleterious effects. In active UC, the normally impenetrable inner mucus layer becomes penetrable, allowing bacteria to reach the epithelium; human tissue and animal data link reduced terminal modifications (incl. sulfation) to barrier failure [8,72]. Inflammation-associated repair programs upregulate epithelial fucosylation by cytokine signaling, as IL-22/STAT3 induces Fut2 expression in intestinal epithelial cells and rapidly enhances α1,2-fucosylated substrates that reshape commensal ecology [73]. In parallel, IL-22 also increases B3GNT7 transcription to promote fucosylation of epithelial glycoproteins, underscoring a direct gene-regulatory link between injury cues and glycosyltransferase expression [51]. Additionally, tumor cells often undergo hypersialylation, overexpressing sialylated antigens such as sialyl-Lewis^x, which promotes immune evasion and metastasis [8]. In conclusion, proper sialylation is critical for forming an effective mucus barrier and maintaining immune homeostasis, while both hypo- and hypersialylation can impair mucosal defense and contribute to disease.

### 2.4. Fucosylation Modification

Fucosylation is a post-translational modification in which L-fucose is added to the terminal positions of mucin O-glycans via α-glycosidic linkages. This modification contributes to the formation of blood group and Lewis antigens. In the colon, fucosylation primarily occurs on the secreted mucin MUC2. This process takes place in the Golgi apparatus, where FUT2 (an α1,2-fucosyltransferase) transfers fucose to the Galβ1-3GalNAc core to generate the H antigen. Subsequently, α1,3/4-fucosyltransferases such as FUT3 and FUT6 add additional fucose residues to form complex Lewis^a, Lewis^b, Lewis^x, and Lewis^y structures [16,74]. Additionally, FUT8 catalyzes the addition of an α1,6-linked fucose to the innermost GlcNAc of N-glycans, and may influence mucin viscoelasticity and barrier properties [75]. Approximately 20% of individuals in Western populations carry loss-of-function alleles of FUT2, known as the “non-secretor” genotype. This genotype leads to the absence of α1,2-fucosylated H antigens in the gastrointestinal mucosa [76]. Compared to secretors, non-secretors have distinct gut microbiota profiles and increased susceptibility to Crohn’s disease and other intestinal disorders [76].

Terminal fucosylation plays dual roles in regulating the mucosal immune barrier and microbial homeostasis. On the one hand, fucosylated mucins serve as adhesion sites and carbon sources for commensal bacteria, functioning as key nutritional substrates. Studies have shown that commensal Bacteroides can metabolize fucosylated glycoproteins secreted by the small intestinal epithelium, thereby supporting microbial stability under nutrient-limited conditions [77]. In the colon, secretors possess an inner mucus layer enriched in α1,2-linked fucose, which promotes colonization by beneficial microbes and enhances resistance to pathogens. Moreover, microbial metabolites—such as aryl hydrocarbon receptor (AhR) ligands and butyrate—upregulate epithelial FUT2 expression, establishing a positive feedback loop that reinforces mucosal fucosylation [78]. On the other hand, during pathogenic invasion. IL-22/STAT3 signaling induces epithelial FUT2-dependent α1,2-fucosylation in vivo and the released fucosylated substrates are consumed by commensals, shifting the community and enhancing colonization resistance; this causal chain is shown with organoids, RNA-seq, and in vivo assays [35,50]. Thus, fucosylation acts both as a commensal-promoting signal and a potential binding site for pathogens. In disease progression, loss of mucin fucosylation compromises mucosal barrier integrity. Studies using FUT2^−^/^−^ (non-secretor) mouse models have an increased susceptibility to intestinal inflammation and exhibit marked shifts in gut microbiota composition [76]. Furthermore, fucosylation is aberrantly upregulated in colorectal cancer, leading to the formation of terminal glycan structures such as sialyl-Lewis^x (sLe^x) and sialyl-Lewis^a (sLe^a), which serve as selectin ligands. These structures facilitate leukocyte extravasation (e.g., of neutrophils and monocytes) and enhance tumor cell adhesion to the endothelium, thereby promoting metastasis [79]. Macrophage-epithelium crosstalk during chronic colitis increases epithelial ST6GALNAC1 expression and drives MUC1-sialyl-Tn (sTn) formation, linking inflammatory damage to sialyltransferase-dependent cancer-associated O-glycan termini [48]. Conversely, enhancing mucus sialylation in vivo preserves inner-layer integrity and reduces colitis, indicating that local sialyltransferase control is a proximal determinant of barrier protection under injury [70]. In summary, balanced fucosylation maintains commensal stability and mucosal homeostasis, whereas. In CRC, aberrant fucosylation yields sLe^x/a selectin ligands that mediate tumor–endothelium adhesion and metastasis; biosynthetic pathways and clinical associations are documented in GI tissues [36].

### 2.5. Sulfation Modification

Sulfation is a post-translational modification in which a sulfate ester group is covalently attached to specific sites on glycans. Although sulfated N-glycans are known in glycoprotein biology, colonic mucus studies consistently demonstrate that sulfation predominantly decorates mucin O-glycans (e.g., GlcNAc-6-O-sulfation), catalyzed by GlcNAc6ST-2/CHST4 in the epithelium [80]. In mucin O-glycans, sulfation typically occurs at the hydroxyl groups of galactose or N-acetylglucosamine (GlcNAc) residues. This reaction is catalyzed by sulfotransferases, which transfer a sulfate group from the activated donor 3′-phosphoadenosine-5′-phosphosulfate (PAPS) to the glycan acceptor [81]. In the intestinal mucosa, multiple sulfotransferases are involved in this process. Among these enzymes, GlcNAc-6-O-sulfotransferase 2 (GlcNAc6ST-2) catalyzes the transfer of sulfate to the 6-position of GlcNAc residues on MUC2 glycans, thereby promoting the production of sulfated mucins in the colon [80]. Colonic mucus generally exhibits a higher level of sulfation than small intestinal mucus. This correlates with the higher microbial density and slower turnover rate of the colonic mucus layer [82]. In patients with ulcerative colitis, mucin sulfation is markedly reduced, potentially due to inflammation-induced downregulation of sulfotransferases or impaired PAPS synthesis and transport. This reduction compromises mucosal barrier function [83].

Loss of sulfomucins is reproducibly observed in ulcerative colitis, and genetic or biochemical disruption of O-glycan sulfation compromises mucus barrier function and exacerbates colitis phenotypes in mice [52] Human studies also show marked decreases in sulfomucins in UC by ^35S-labeling/chemical assays, supporting a barrier-relevant role for sulfation; reduced sulfation associates with inflammation and impaired mucus properties [37]. The highly negative sulfate groups form ionic interactions with positively charged amino acids in the mucin backbone, enhancing gel cross-linking and mechanical resilience [7,84]. Access to highly sulfated colonic O-glycans requires bacterial sulfatases; loss or inhibition of these enzymes reduces utilization of colonic mucin glycans, demonstrating that sulfation impedes enzymatic access and slows degradation [41]. Consequently, higher levels of sulfation slow microbial degradation and help maintain the integrity of the mucus barrier [85]. In the healthy colon, sulfation and sialylation together confer a strong negative charge, contributing to bacterial repulsion and reducing enzymatic erosion of the mucus. In disease states, altered sulfation patterns weaken this defense. For example, mouse models deficient in PAPS synthase 2 exhibit reduced mucin sulfation, increased microbial infiltration, and heightened inflammatory responses [74]. In addition, terminal sulfate groups can form specialized glycan ligands involved in immune modulation. To summarize, adequate sulfation enhances the protective properties of the mucus layer and increases its resistance to degradation, whereas reduced sulfation increases susceptibility to microbial invasion and inflammation.

## 3. Regulation of Intestinal Barrier Homeostasis by Mucin Glycosylation Modifications

**Figure 2:**
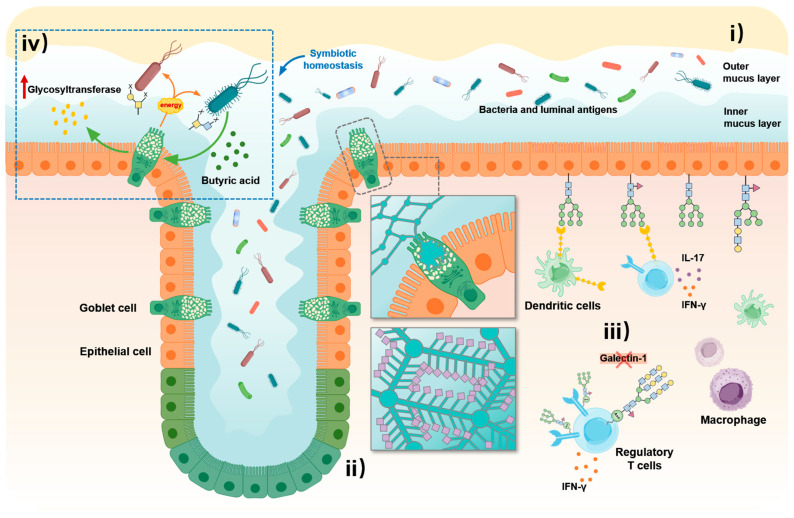
Role of Mucin Glycosylation in Maintaining Intestinal Barrier Homeostasis. (**i**) Under physiological conditions, fully glycosylated mucins form a bilayer mucus barrier on the intestinal surface. The inner mucus layer is dense and sterile, effectively preventing pathogen access to epithelial cells, whereas the outer mucus layer is more loosely structured, allowing colonization and proliferation of commensal bacteria. (**ii**) This spatially differentiated structure constitutes an efficient physical barrier, preventing pathogen invasion and finely regulating the luminal microenvironment to maintain microbial homeostasis. (**iii**) Additionally, abundant glycans within the mucus serve not only as adhesion sites and energy sources for commensal microbes but also specifically interact with immune cell receptors such as Siglecs and lectins (e.g., Galectin-1). These interactions modulate the activity of innate immune cells, including dendritic cells and macrophages, thereby inducing and maintaining mucosal immune tolerance and suppressing excessive inflammatory responses. (**iv**) Meanwhile, microbial metabolites, particularly short-chain fatty acids like butyrate, can upregulate the expression of glycosyltransferases in host epithelial cells, further enhancing mucin glycosylation. This forms a positive feedback loop that reinforces the homeostasis of the intestinal barrier.

The intestinal barrier is a multi-layered defense system comprising physical, immune, and microbial components, which collectively maintain colonic homeostasis [6]. MUC2-stratified inner/outer layers, SIgA–mucus anchoring, and commensal PUL-mediated glycan foraging constitute experimentally defined cooperative mechanisms across the barrier [86]. For example, highly glycosylated mucins form a two-layered colonic mucus that blocks pathogens and regulates the luminal distribution of water, ions, and immune mediators (e.g., antimicrobial peptides and IgA) [7,87]. The mucosal immune system resides within the barrier and maintains a balance between antigen recognition and immune tolerance [88]. Meanwhile, the commensal gut microbiota colonizes the outer mucus layer, establishing a mutualistic relationship with the host [89]. Defined mucin glycan features (e.g., MUC2-dependent stratified mucus, SIgA–mucus anchoring, and commensal PUL-mediated foraging) have experimentally demonstrated roles across the physical, immune, and microbial layers of the barrier [86]. These modifications not only serve as structural components of the physical barrier but also act as immune recognition signals and microbial attachment sites. Through these roles, mucin glycans help maintain barrier integrity and ensure a properly balanced immune response under homeostatic conditions (Figure 2).

### 3.1. Effects on the Physical Barrier

The colonic epithelium is covered by a two-layered mucus barrier composed primarily of mucins. The inner mucus layer is dense and firmly adherent to the epithelial surface. Under homeostatic conditions, the MUC2-organized inner colonic mucus layer is largely devoid of bacteria, whereas the outer layer is colonized by microbes [86]. In contrast, the outer mucus layer is loosely structured and can be colonized by the commensal gut microbiota [90]. For the mucus layers to form and renew, goblet cells must continuously secrete highly O-glycosylated gel-forming mucins such as MUC2. The extensive glycosylation of these mucins is critical for their ability to maintain the physical barrier function of the mucus layer. First, the abundant O-glycan chains (accounting for ~50–80% of a mucin’s mass) impart high water solubility and an expanded conformation to mucins, allowing them to rapidly absorb water and swell after secretion to form a gel-like mesh network [91,92]. In goblet cells, glycosylated mucins are packaged and condensed in secretory granules under conditions of high calcium and low pH. This microenvironment helps keep the mucin condensed and maintains its stability in the unsecreted state. Once mucin is secreted into the intestinal lumen, Ca^2+^ is chelated and the pH is neutralized. Upon secretion, chelation of Ca^2+^ and neutralization of pH trigger MUC2 unpacking and layer formation; EM/biophysical data demonstrate Ca^2+^/pH-dependent packing-release rather than a fixed ‘several-hundred-fold’ expansion [85,91]. Moreover, the O-glycosylation profile of mucins determines the stratification and renewal dynamics of the mucus layer. O-glycans with core-1 and core-3 structures have been shown to be essential for maintaining the integrity of colonic mucus. In mice lacking these glycans, the mucus layer becomes thinner and is easily penetrated by bacteria [7,33]. The inner mucus layer in the colon is typically 50–150 µm thick [82,93,94]. The outer mucus layer is more loosely organized, with larger pores that can accommodate particles of around 0.5 µm. It is continuously “brushed” outward and renewed by intestinal peristalsis and ongoing secretion. Peristalsis and ongoing MUC2 secretion convert inner to outer mucus and shed bound microbes/particles, limiting epithelial contact by bacteria [60,86,95]. Therefore, normal mucin glycosylation ensures the structural integrity and continuous renewal of the mucus barrier. This allows the physical barrier to effectively protect the intestinal epithelium from mechanical damage and microbial intrusion.

The physicochemical properties of mucus are also finely tuned by mucin glycosylation. The abundant glycan chains of mucins impart an extremely high water-holding capacity and a gel-like viscoelasticity to the mucus, thereby allowing the mucus layer to form a lubricating and stable gel barrier in the intestinal lumen [96]. Among these properties, terminal glycan modifications such as sulfation and sialylation introduce negatively charged groups. These negative charges enhance the mucus’s ability to bind water molecules and increase repulsive forces within the gel, which helps maintain an appropriate viscosity and thickness [85,97]. In addition, mucin glycosylation increases the mucus layer’s resistance to enzymatic degradation through steric hindrance. For example, O-glycosylation at specific sites on the MUC2 mucin can prevent proteases such as pancreatic elastase from cleaving the mucin, thereby preserving the integrity of the mucus layer [77]. Negatively charged mucins electrostatically bind cationic AMPs and can sequester microbial factors, contributing to adhesion interference and localized antimicrobial activity [98]. Pathogenic microbes secrete mucus-degrading enzymes that often must remove specific terminal residues from mucin glycans before they can reach the protein core. Under healthy conditions, the mucus is rich in diverse glycan structures, making this process slow and inefficient [35,77]. At the level of secretory control/regulation, goblet-cell mucus exocytosis requires a calibrated ER-unfolded protein response that enables rapid MUC2 condensation–expansion cycles during secretion, thereby preserving gel architecture under physiological flow [99]. Golgi homeostasis further shapes mucin glycan end-groups, as alterations in luminal pH or oxygen sensing mislocalize glycosyltransferases and remodel terminal modifications, with consequent effects on hydration, charge density, and proteolytic resistance of the mucus [29]. Overall, normal mucin glycosylation endows the mucus layer with excellent water retention and viscoelastic properties. It provides a natural barrier against digestive enzymes and microbial invasion, ensuring that the physical barrier functions optimally under physiological conditions.

### 3.2. Effects on the Immune Barrier

The intestinal mucosal immune barrier consists of various resident immune cells in the lamina propria and epithelium (such as macrophages, dendritic cells, and regulatory T cells (Tregs)) as well as secreted immune factors (such as secretory IgA (SIgA), interleukin-10 (IL-10), and antimicrobial peptides). Its homeostasis relies on a dynamic balance between immune tolerance toward beneficial commensals and rapid immune activation against potential pathogens [100]. The extensive glycosylation of intestinal mucins (e.g., MUC1, MUC2, MUC3) not only affects their gel structure and barrier function but also contributes to immune recognition between commensals and epithelial cells through their glycan patterns, thereby playing a key role in mucosal immune tolerance and the regulation of inflammation [101]. On the one hand, the host’s own glycan structures function as “self-associated molecular patterns,” enabling the innate immune system to distinguish self from non-self and thus avoid excessive reactions against normal tissues and commensal bacteria [102]. Glycoantigens present on the intestinal epithelium and in mucus (such as blood group antigens and Lewis antigens) bind to pattern recognition receptors or lectin receptors on immune cells, delivering “safe” signals that promote the maintenance of mucosal tolerance [103]. On the other hand, the mucus glycan network also participates in antigen capture and presentation. Mucus-associated SIgA and epithelial sampling pathways (goblet-cell) concentrate antigens and promote immune exclusion/tolerogenic presentation [104]. This process helps induce non-inflammatory immune responses, such as IgA production [6]. Under healthy conditions, mucin glycosylation regulates the colon’s antigen sampling process (mediated by goblet cells and M cells), ensuring that the mucosal immune system mounts appropriately scaled responses to luminal antigens without causing tissue damage [105]. This glycan-dependent immune recognition mechanism similarly applies to the functional positioning of mucosal immune effectors. For example, the secretory component (SC) of SIgA contains abundant glycans. When SIgA is transcytosed across the epithelium via the polymeric Ig receptor, the SC remains attached and binds to the mucus through its glycans, thereby anchoring IgA in the mucus layer [104]. This mechanism localizes IgA to the mucosal surface to exert immune exclusion. SIgA associated with the mucus can trap and neutralize pathogenic toxins invading the mucosa without activating complement, thereby preventing inflammatory damage. The mucus glycan network also concentrates various mucosal antimicrobial substances. For instance, positively charged antimicrobial peptides can adsorb to negatively charged mucin glycans, forming a highly concentrated, localized antimicrobial barrier in the mucus [106]. Epithelial and mucus sialylated glycans engaging inhibitory Siglecs and SIgA anchoring in mucus are documented mechanisms that raise activation thresholds yet enable immune exclusion [107].

Mucosal glycans also function as key signaling molecules that regulate the functional state of immune cells. For instance, Sialic-acid/Siglec interactions on neutrophils/macrophages engage ITIM signaling and dampen activation in vivo/in vitro, providing a defined inhibitory pathway [107]. For example, when Siglec-9 on neutrophils and macrophages binds to sialic acid-rich glycans on epithelial cells or in the mucus, it triggers inhibitory signaling pathways. This in turn reduces the activation of these cells and prevents excessive inflammatory responses that could damage tissues [71,108]. Glycan-lectin interactions are also an important aspect of mucosal immune regulation. The galectin family of proteins can recognize β-galactose-terminated glycans in the mucosa and mediate various immunomodulatory effects [109]. For instance, when Galectin-1 binds to glycosylated ligands on T cells, it can induce apoptosis or functional inactivation. Consequently, activated effector T cells undergo increased apoptosis, while regulatory T cells exhibit enhanced activity, producing an immunosuppressive effect in the mucosal environment [110,111]. In the colonic mucosa, endogenous Galectin-1 has been shown to limit the intensity of inflammatory responses, whereas its absence leads to excessive T cell proliferation and elevated levels of the pro-inflammatory cytokine IFN-γ [111]. Additionally, C-type lectin receptors on dendritic cells and macrophages (such as DC-SIGN) can recognize specific fucosylated or mannosylated glycans. This recognition affects antigen presentation and cytokine production, thereby shaping the direction of mucosal immune responses [102,112]. Epithelial intracellular O-GlcNAcylation functions as a nutrient- and microbiota-responsive integrator of mucosal signaling, where genetic depletion of O-GlcNAc transferase (OGT) in intestinal epithelium compromises barrier homeostasis and increases susceptibility to experimental colitis [113]. Mechanistically, O-GlcNAcylation fine-tunes canonical pathways that set mucosal immune tone, including NF-κB and type-2 STAT6 signaling, thereby shaping epithelial alarmin programs, goblet-/tuft-cell responses, and downstream effector deployment [114]. Pharmacologic augmentation of epithelial O-GlcNAcylation by O-GlcNAcase inhibition mitigates chemically induced colitis in vivo, underscoring its tractability as a mucosal-homeostasis node [115]. Overall, through interactions with lectin receptors, sialylated epithelial/mucus glycans engage inhibitory Siglecs on myeloid cells, and fucosylated Lewis antigens are recognized by DC-SIGN, providing defined lectin-glycan checkpoints that calibrate mucosal responses [107]. Commensals adhere via defined glycan motifs (e.g., fucose/galactose-binding adhesins) and forage mucin O-glycans via PUL-encoded glycosidases; fiber deprivation shifts the community toward mucus erosion [116], balancing protective immunity and immune tolerance while maintaining the functional stability of the immune barrier.

Besides, within the intestinal epithelium, mucin glycosylation can constitute a self-associated molecular pattern (SAMPs) system that calibrates host-microbe crosstalk and sustains immune tolerance [117]. At the receptor level, sialylated O-glycans on epithelial mucins engage inhibitory Siglecs and trigger ITIM-dependent signaling, thereby elevating activation thresholds and maintaining mucosal tolerance [118]. In the gut, maintaining mucus sialylation preserves the stratified inner layer, supports commensal residency and metabolite balance, and ameliorates intestinal inflammation [70]. In parallel, fucosylated Lewis antigens displayed on epithelial mucins provide a defined glycan-lectin interface with DC-SIGN on myeloid cells, supplying a structural basis for tolerogenic modulation at the mucosal surface [119]. Beyond its biophysical gel properties, the secreted mucin MUC2 delivers tolerogenic cues to intestinal dendritic cells in vivo, reinforcing oral tolerance to luminal antigens [120]. Moreover, galectin-3 differentially “reads” epithelial mucin O-glycan profiles, demonstrating that host endogenous lectins decode defined glycan patterns to shape antigen-presenting-cell behavior [109]. Field syntheses further consolidate that epithelial/mucin glycosylation programs regulate immune activation and tolerance in the intestine and that inflammatory remodeling of glycosylation contributes to pathobiology [16]. Accordingly, truncation or remodeling of epithelial mucin O-glycans weakens these lectin-mediated checkpoints and predisposes to chronic mucosal inflammation, underscoring the SAMPs function of mucin glycosylation in gut homeostasis [121].

### 3.3. Effects on the Gut Microbiota

The colonic mucus layer serves not only as a physical barrier but also as an ecological niche for symbiosis between the host and the microbiota [122]. Glycan chains on mucins serve both as "anchors" for microbial attachment and as nutrient sources, thereby selectively influencing the composition and spatial distribution of the gut microbiota [123]. Under healthy conditions, numerous commensal bacteria colonize the outer mucus layer, and many of these strains can specifically recognize and utilize host mucin glycans [34]. For example, exposed terminal glycans such as fucose and galactose in the mucus can be recognized by binding proteins on the surface of Bacteroides and Lactobacillus species, thereby promoting their attachment to the mucus matrix [124,125]. This adhesion helps commensal bacteria colonize the gut and thereby prevents pathogens from occupying those niches. In addition to serving as attachment sites, host glycans are also important metabolic substrates for many gut commensals. Mucin O-glycans contain abundant monosaccharides such as N-acetylgalactosamine, galactose, fucose, and sialic acid, which can be progressively broken down and utilized by glycosidases from the commensal microbiota [7,35]. The mucus layer provides a continuous, low-level source of carbon for mucosal symbionts. For instance, mucus-resident Akkermansia and various Bacteroides species can degrade mucin glycans to obtain energy, allowing them to thrive within the host [7,22]. The host also supports the growth of beneficial commensals by providing glycans in the mucus. Studies have shown that when dietary fiber is insufficient. During fiber deprivation, the microbiota reproducibly increases mucus-glycan foraging, thinning the inner layer and increasing epithelial proximity and inflammation [116]. Different microbes vary in their ability to utilize specific glycan structures. Therefore, the composition of the host’s mucus glycome largely determines the makeup of the gut microbiota [6,41]. In summary, normal glycosylation provides attachment sites and metabolizable substrates that create an ecological environment conducive to commensal colonization and cross-feeding. In turn, this strengthens the gut microbial barrier’s defense against pathogens and supports its role in the host’s nutrient metabolism.

Notably, the gut microbiota and the host’s glycosylation exhibit a bidirectional dynamic interaction. Commensal bacteria not only passively adapt to the glycan niches provided by the host, but also actively regulate the host’s glycosylation patterns through feedback mechanisms to maintain symbiotic homeostasis [102]. One example of such a feedback mechanism is the induction of fucosyltransferase expression in epithelial cells by commensal bacteria. Studies have found that when certain Bacteroides species colonize the small intestine, they stimulate host epithelial cells via microbial-associated molecular patterns to secrete cytokines that promote secretory IgA production and to upregulate FUT2 gene expression. This causes the epithelium and mucus to produce more fucosylated glycans for the commensals to utilize [124,126]. This feedback mechanism also operates in the colon. Germ-free animals have a mucus O-glycosylation profile that differs significantly from that of conventionally raised animals. Upon colonization with commensal microbes, the host adjusts the expression of multiple glycosyltransferases, thereby creating a glycan environment more suitable for the microbiota [41,127]. Besides, dietary fiber deprivation reprograms the microbiota toward mucus-glycan foraging, thins the inner mucus layer, and increases epithelial proximity and inflammation, demonstrating condition-dependent erosion of the barrier [128]. Human mucin-degrading taxa encode polysaccharide utilization loci (PULs) and a stepwise armamentarium of glycoside hydrolases and sulfatases that deconstruct mucin O-glycans in vivo [116]. Prominent community sialidases desialylate terminal mucin glycans and expose underlying cores, facilitating further enzymatic access and altering gel properties at the epithelial interface [129]. Defined mucin epitopes, including Lewis antigens and terminal sialyl-/fucosyl- motifs, provide adhesion sites and carbon sources for taxa such as Akkermansia muciniphila, thereby shaping niche residency in human cohorts. Meanwhile, the gut microbiota have been suggested to downregulate glycosylation at specific sites on mucins. This alters mucus viscosity and degradability to balance microbial utilization of mucus with the preservation of mucus integrity [22]. Under homeostatic conditions, the host and microbiota “communicate” through glycosylation-mediated signals. Molecules produced by commensal metabolism (such as short-chain fatty acids) can also influence the expression of genes for glycosylation enzymes in epithelial cells, as well as mucin secretion [112]. For instance, butyrate has been shown to cause colonic epithelial cells to increase glycosyltransferase expression and mucin synthesis, thereby strengthening the mucus barrier as a feedback adaptation to the microbiota’s metabolism [130,131]. This bidirectional regulation between the host and microbiota via glycosylation ensures that the mucus barrier provides a stable niche for commensals without becoming excessively depleted. In this way, microbial diversity and homeostasis are maintained. Therefore, through fine coordination between the different components of the intestinal barrier and the gut microbiota, glycosylation modifications achieve a dynamic equilibrium between the host and microorganisms. This equilibrium provides continuous support for gut health.

## 4. Methods for the Detection and Analysis of Mucin Glycosylation Modifications

As described earlier, mucin glycosylation modifications have significant impacts on physiological functions and disease development. Abnormal mucin glycosylation patterns can also serve as potential biomarkers during the progression of colonic diseases [132,133]. However, the long protein backbone of mucins, their high viscosity, and the diversity of glycan types present significant challenges for traditional analytical methods [134,135]. In the following sections, detection and analysis methods for mucin glycosylation modifications are presented in three main categories: histochemical staining, mass spectrometry analysis and imaging, and fluorescent labeling and imaging techniques.

### 4.1. Histochemical Staining

Early studies primarily relied on histochemical staining to visualize mucin distribution. For example, Alcian Blue (AB) at pH 2.5 stains acidic mucopolysaccharides blue (e.g., those containing sialic acid or sulfate groups), whereas it does not stain neutral mucopolysaccharides [135,136]. AB is typically used in combination with Periodic Acid-Schiff (PAS) staining. PAS stains neutral mucopolysaccharides pink, thus distinguishing acidic and neutral mucins in the same sample. Vadivazhagan et al. utilized combined AB-PAS staining with digital image analysis to quantitatively compare the levels of acidic and neutral mucins in colon cancer tissues [136]. In addition, lectin and monoclonal antibody staining targeting specific glycan epitopes can provide additional information for glycan typing. For example, fluorescent or biotin-labeled lectins (such as PNA, which binds Galβ1-3GalNAc, or UEA-I, which binds α1-2 fucose) can bind to specific glycan chains on mucins and are used to qualitatively detect their distribution in tissues [137]. Similarly, specific monoclonal antibodies (e.g., against the Tn antigen or the CA19-9 antigen) have been used in immunohistochemical staining to detect abnormal mucin glycosylation in cancer tissues [138]. However, these methods do not provide precise information on monosaccharide composition or glycan structure, and thus serve only as rapid screening tools.

### 4.2. Mass Spectrometry Analysis and Mass Spectrometric Imaging

Mass spectrometry is a powerful tool for deciphering the structural composition of the mucin glycome. Common approaches include releasing mucin O-glycans by chemical or enzymatic methods (for example, via reductive β-elimination) and then derivatizing the released glycans (for instance, by permethylation or DMB labeling), followed by analysis using matrix-assisted laser desorption/ionization time-of-flight mass spectrometry (MALDI-TOF MS) [133,139]. In addition, released glycans can be separated by liquid chromatography (LC) and detected via fluorescence or mass spectrometry, allowing accurate identification of glycan composition, branching patterns, and specific modifications (such as sialylation or fucosylation) [132,133]. Progress has also been made in direct sequencing techniques that do not require prior glycan release. For example, by enriching mucins or digesting glycopeptides and then performing LC-MS/MS, it is possible to obtain information on both the mucin protein backbone and its attached glycans simultaneously [132,134]. Mahoney et al. summarized strategies for mucin analysis by mass spectrometry, including specific enrichment and digestion steps, and noted that the dense glycosylation of mucins can hinder enzymatic digestion and ionization. As a result, optimized sample preparation and data analysis methods are required [134].

Mass spectrometry imaging (MSI) allows direct mapping of glycosylated molecules in tissue sections. Studies have applied MALDI-MSI to analyze the spatial distribution of mucin O-glycans in colon tissue [139], as well as the distribution of N-glycan types [140]. In summary, chromatography-coupled mass spectrometry and imaging techniques provide highly sensitive approaches for both qualitative and quantitative analysis of mucin glycosylation. Nonetheless, issues such as high sample complexity and difficulty in distinguishing glycan isomers still persist [133,134].

### 4.3. Fluorescent Labeling and Imaging Methods

Fluorescence-based chemical labeling and imaging techniques can be used to visualize the dynamic distribution of mucin glycosylation in living cells or tissues. For example, modified monosaccharide precursors (such as peracetylated N-azidoacetylgalactosamine, Ac_4GalNAz) can be introduced into cells or animals via metabolic labeling [141]. These azide-containing sugars are metabolically incorporated into mucin O-glycans in vivo. Subsequent bioorthogonal “click” chemistry (e.g., Staudinger ligation or copper-catalyzed cycloaddition) attaches a fluorescent probe to the incorporated Ac_4GalNAz groups, thereby allowing direct visualization of newly synthesized mucins under a microscope [142,143]. Another strategy is to pretreat the tissue with a specific mucinase that recognizes and cleaves mucin domains. Fluorescent probes are then coupled to the cleaved oligosaccharide stubs, achieving amplified and specific visualization of mucins in situ [144,145]. Additionally, fluorescently labeled lectin or antibody probes can bind directly to specific glycans in cells or tissue samples, thereby enabling localization of glycosylation at the cellular level [146,147]. These methods can be applied in live imaging and in tissue section analysis, providing dynamic or even subcellular-resolution information on the distribution of mucin glycosylation. It should be noted that metabolic labeling is limited by cellular uptake efficiency and metabolic pathway constraints, whereas lectin- or antibody-based probes require careful control of specificity and background signal [110,148]. Nevertheless, fluorescence-based labeling techniques offer valuable tools for investigating the spatial distribution and biological functions of mucin glycosylation.

## 5. Abnormal Mucin Glycosylation in Common Colonic Diseases and Associated Intervention Targets

### 5.1. Ulcerative Colitis (UC)

UC is a chronic, relapsing inflammatory disease of the colonic mucosa and is a major form of inflammatory bowel disease (IBD). Pathologically, it is characterized by diffuse mucosal inflammation, superficial ulcers, and crypt abscesses. Clinically, UC presents with recurrent diarrhea and purulent, bloody mucus in stool, among other symptoms [149]. In 2017, approximately 6.8 million people worldwide had IBD, the vast majority of whom were UC patients, and the incidence of IBD continues to rise [150]. Recent studies have found that glycosylation of major mucins (such as MUC2) in the colonic mucus layer of UC patients is significantly abnormal. Specifically, O-glycan structures are truncated, resulting in reduced carbohydrate content in mucin extracts and a markedly shorter average O-glycan chain length [16,151]. In addition, [^35S] sulfate labeling studies confirmed that rectal mucus sulfation is significantly decreased in UC patients [52]. These glycosylation abnormalities severely compromise the integrity of the mucus barrier. In healthy colon the inner mucus layer is impervious to bacteria, as shown by microscopy and bacterial exclusion assays [94]. However, in UC and corresponding animal models, this inner layer is often penetrated by bacteria [37]. As truncated and desulfated mucin molecules are more easily degraded by the gut microbiota, mucin-degrading bacteria (such as Bacteroides species) can dominate and overgrow, further destroying the mucus barrier [152,153]. As a consequence, exposed mucin epitopes or newly generated neo-antigens may induce immune responses, worsening intestinal inflammation and creating a vicious cycle of dysbiosis and immune imbalance [154,155].

**Figure 3:**
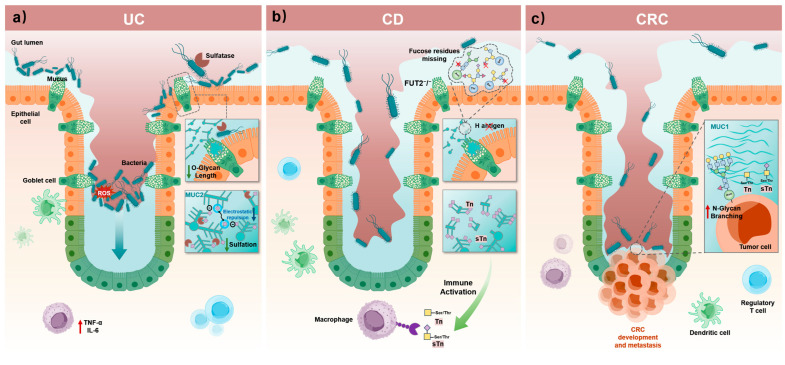
Aberrant Characteristics of Mucin Glycosylation in UC, CD, and CRC. (**a**) In ulcerative colitis (UC), decreased mucin secretion leads to significant thinning of the mucus layer, accompanied by truncation of glycan chains and reduced terminal sulfation levels. These changes disrupt the physical barrier function of the mucus, increasing pathogen penetration risk, triggering excessive immune responses, and exacerbating chronic intestinal inflammation. (**b**) In Crohn’s disease (CD), mucins exhibit pronounced deficiency in fucosylation, along with increased expression of abnormal, immature glycans such as Tn/sTn antigens. These abnormalities not only compromise barrier integrity but also directly engage lectin-like receptors on immune cells, activating pro-inflammatory Th1/Th17 pathways. (**c**) In colorectal cancer (CRC), membrane-bound mucins such as MUC1 are aberrantly overexpressed, featuring shortened and structurally simplified glycans. These abnormal modifications severely impair mucosal barrier function and expose cryptic glycan epitopes, aiding tumor cell evasion of host immune surveillance. Moreover, increased branching of N-glycans in tumor cells promotes altered intercellular adhesion and aberrant signal transduction, enhancing invasive and metastatic potential.

Given these pathological mechanisms (Figure 3a), restoring normal glycosylation is a promising therapeutic strategy. Animal studies have demonstrated that L-fucose supplementation (aimed at increasing MUC2 terminal fucosylation) can significantly alleviate experimental colitis in mice [156]. In terms of drug delivery, strategies using glycan ligands to target inflamed tissue have also shown progress. For example, dexamethasone nanoparticles decorated with a peanut agglutinin (PNA) tetramer (which binds the T-antigen) specifically targeted inflamed intestinal segments and significantly reduced pathological damage [157,158]. Moreover, approaches to modulate the gut microbiota in order to restore a normal glycosylation environment have been explored. Probiotics such as Lactobacillus and Bifidobacterium can stimulate intestinal mucin secretion and inhibit pathogenic bacterial adhesion [159]; Furthermore, a probiotic mixture (VSL#3) has been shown to maintain remission in UC patients by promoting mucus secretion [160,161]. Similarly, strategies to inhibit mucin glycan degradation have also proven effective. For instance, oral sialidase inhibitors can reduce the overgrowth of pathogenic E. coli and lessen the severity of colitis [162]. In summary, preclinical studies show that modulating terminal glycan features (e.g., enhancing fucosylation or preserving sialylation) can strengthen the mucus barrier and mitigate colitis in mice, nominating epithelial glycosylation as a tractable therapeutic axis [71].

### 5.2. Crohn’s Disease (CD)

CD is a chronic, relapsing inflammatory bowel disease that can affect the entire gastrointestinal tract, and its pathogenesis is closely related to mucosal barrier function [163]. In CD patients, mucosal glycosylation patterns are markedly altered. In IBD tissues, mucins show decreased sulfation by [^35S] labeling/chemical assays and reduced sialylation; in CD, fecal mucinomics reveals significantly fewer sialylated O-glycans relative to controls, changes that accompany active inflammation [164]. FUT2 non-secretor status (loss of epithelial H-antigen) shows genome-wide significant association with CD in Europeans and East Asians [126]. In healthy colon, a MUC2-organized two-layer mucus system features a dense inner layer devoid of bacteria and a looser outer layer colonized by microbes; barrier failure allows bacterial approach to the epithelium [94]. However, in CD, defects in O-glycosylation compromise this mucus barrier. Mouse models have shown that complete loss of core 1- and core 3-derived O-glycans (core 1/3 O-glycosylation) causes the mucus barrier to collapse and triggers bacteria-driven inflammasome activation, leading to spontaneous colitis and colonic carcinoma [40]. Combined genetic models demonstrate causality: mice lacking intestinal core 1- and core 3-derived O-glycans develop a penetrable inner mucus layer, spontaneous colitis, and increased cancer risk, supporting a direct contribution of epithelial O-glycan loss to colitis pathogenesis [33]. Human genetics and experimental models converge to implicate epithelial glycosylation pathways—e.g., FUT2-dependent α1,2-fucosylation and core 1/3 O-glycans—in barrier integrity and microbial containment; their disruption increases CD risk or promotes colitis phenotypes [126]. Clinical analyses have also identified specific glycosylation changes in the CD mucosa. For example, in CD patients, the MUC1 glycoprotein is overexpressed at inflammatory lesion sites but is hypoglycosylated [165], This may increase mucosal permeability and activate immune-inflammatory pathways. A recent fecal glycomics study showed that sialylation of excreted mucins is significantly reduced in CD patients [166], suggesting that mucins with reduced sialylation could serve as a potential non-invasive biomarker for CD. O-glycans in the CD mucosa often show increased expression of truncated antigens such as the T antigen and sialyl-Tn (sTn) antigen [16], as well as fucosylated structures with increased co-expression on immunoglobulins. These abnormal glycans can interact with host lectins (such as selectins and galectins), affecting inflammatory cell recruitment and cancer risk [16,40]. In summary, the development of Crohn’s disease is closely linked to abnormal O-glycosylation of the mucus layer. These glycosylation changes undermine the stability of the intestinal mucosal barrier and exacerbate the microbiota-immune imbalance (Figure 3b).

In recent years, intervention strategies targeting aberrant glycosylation in CD have gained increasing attention. Researchers have attempted to restore the damaged mucus barrier by supplementing glycan precursors or modulating intestinal metabolites. Oral or enteral supplementation with N-acetylglucosamine (GlcNAc), a glycosaminoglycan precursor, has been administered to patients. Clinical data indicate that this treatment improved symptoms in refractory IBD and increased the mucopolysaccharide content of intestinal mucus [167]. Its molecular mechanisms include the inhibition of Th1/Th17 immune responses and the promotion of mucosal healing [168]. In addition, dietary interventions that promote the metabolism of beneficial microbiota can positively influence mucin glycosylation. Short-chain fatty acids (e.g., butyrate) produced by dietary fiber fermentation can upregulate glycosyltransferase expression in mucus-secreting cells [130]. This upregulation enhances mucin sulfation and fucosylation at the molecular level. Animal experiments have shown that a high-fiber diet combined with Bifidobacterium supplementation significantly preserves mucus layer thickness, preventing excessive mucus degradation by the microbiota [116,169]. Blocking bacterial mucin-degrading enzymes is another potential strategy. However, due to the diversity and non-specificity of microbial polysaccharidases, the safety and efficacy of this approach have yet to be established. Furthermore, forward-looking approaches have been proposed, such as supplementing exogenous polysaccharides to coat the mucosa or using genetic engineering to express missing glycosylation enzymes (such as core 3 N-acetylglucosaminyltransferase) in the intestine to repair the mucus barrier [170,171]. Colonic inflammation in Crohn’s disease also associates with elevated epithelial O-GlcNAcylation that potentiates NF-κB activation and restrains autophagy, providing a mechanistic link between intracellular glycosylation dynamics and chronic mucosal inflammation [172]. These observations nominate epithelial O-GlcNAc cycling as a complementary target alongside mucin-centric strategies in CD. Overall, indirectly enhancing mucin glycosylation through nutritional supplementation, probiotic and prebiotic interventions, and metabolic regulation, as well as developing small-molecule or gene therapies directly targeting core glycosylation enzymes, represent promising but still exploratory directions for glycosylation-targeted interventions in CD.

### 5.3. Colorectal Cancer (CRC)

In CRC, mucin glycosylation undergoes significant abnormalities that drive tumor development and progression. In normal colonic epithelium, the predominant secreted mucin is MUC2, which forms a thick mucus layer that blocks bacteria [86]. However, in many colonic adenomas and adenocarcinomas, MUC2 expression is diminished or even lost, exposing the intestinal lumen to inflammatory and tumor-promoting factors [173]. Experiments in mice have shown that the loss of Muc2 can lead to the spontaneous formation of colonic adenomas [174]. This finding further confirms the importance of an intact mucus barrier in suppressing tumor development. Meanwhile, tumor cells often express higher levels of membrane-bound mucins (such as MUC1), and their O-glycan biosynthetic pathways are reprogrammed to produce shorter glycan chains [175]. Classic tumor-associated O-glycans such as the T antigen, Tn antigen, and sTn antigen are markedly upregulated. These abnormally truncated glycans, once exposed, can promote cancer cell adhesion, invasion, and immune evasion (Figure 3c). For example, multiple studies have noted that dysregulated expression of the core 1 β1,3-galactosyltransferase (C1GALT1) and its chaperone Cosmc in CRC causes O-glycan chains to terminate at the Tn antigen precursor stage [176]. Additionally, overexpression of N-acetylglucosaminyltransferase V (GnT-V) leads to the formation of highly branched N-glycans and promotes the addition of polysialic acid chains. These changes collectively alter cell-cell adhesion and signal transduction, thereby increasing the invasive and metastatic potential of tumor cells [177]. Taken together, these abnormal glycosylation modifications in cancer cells (including increased sialylation and fucosylation) provide tumor cells with the capabilities to adhere to vascular endothelium, evade immune surveillance (e.g., through interactions with colitis-associated bacteria and galectins), and promote metastasis [178,179]. Core-pathway remodeling accompanies tumorigenesis, as B3GNT6 (core-3 transferase) is frequently downregulated in CRC and associates with poor outcomes, consistent with a loss of protective core-3 O-glycan structures [46]. Emerging data further suggest a functional B3GNT6-MUC2 axis in colitis-to-cancer progression, highlighting inflammation-driven reprogramming of mucin-type O-glycan cores [180]. Overall, colorectal cancer cells reprogram mucin glycosylation so that tumor-associated carbohydrate antigens become dominant while the protective mucus barrier is weakened. These changes play crucial roles in tumor progression.

Intervention strategies targeting glycosylation abnormalities in CRC mainly focus on immunotherapy and enzyme inhibition. One approach is to utilize tumor-specific carbohydrate antigens as targets for vaccines or antibody therapies. For example, an sTn-keyhole limpet hemocyanin (KLH) conjugate vaccine (Theratope) was tested in clinical trials to induce an anti-sTn antibody response [181]. In addition, monoclonal antibodies and engineered lectins have been developed to target tumor-associated glycoantigens such as Tn, sTn, and sialyl-Lewis antigens. Preclinical models have shown that these agents can inhibit CRC cell invasion and metastasis [182,183]. Another approach is to directly inhibit the enzymes involved in aberrant glycosylation. For instance, the systemic α1,2-fucosylation inhibitor SGN-2FF effectively inhibited glycoprotein fucosylation and showed preliminary anti-tumor activity in a Phase I study. However, this trial was terminated due to toxic effects such as thrombosis [184]. Similarly, a small-molecule inhibitor of the core fucosyltransferase FUT8, FDW028, showed potent anti-tumor effects in a CRC animal model [185]. This finding indicates that disrupting core fucosylation in tumor cells can enhance immune recognition and suppress tumor growth. Therefore, immunotherapies targeting tumor-specific O-glycans and inhibitors of glycosylation enzymes represent promising therapeutic avenues for CRC. These strategies are expected to be combined with existing treatments to improve patient outcomes.

## 6. Conclusions

To summarize, mucin glycosylation plays a pivotal role in maintaining intestinal barrier integrity and homeostasis. Glycosylation not only determines the physical and biochemical properties of the mucus layers but also contributes to the dynamic regulation of the immune and microbial barriers. These processes ensure effective protection of the intestinal epithelium from microbial invasion and excessive immune activation. Abnormal mucin glycosylation is closely associated with colonic diseases such as ulcerative colitis, Crohn’s disease, and colorectal cancer. Such aberrant glycosylation significantly impairs barrier function and exacerbates disease progression. Current methods for analyzing mucin glycosylation include histochemical staining, mass spectrometry, and lectin- or antibody-based fluorescence labeling. These approaches have provided essential insights into mucin glycan structures and their pathological alterations. Importantly, accumulating evidence indicates that intracellular glycosylation in epithelial cells functions as a central regulator of barrier-relevant signaling and cellular homeostasis in the healthy intestine. This regulatory layer includes ER and Golgi quality-control systems that support mucin folding and secretion, and cytosolic or nuclear O-GlcNAc signaling that integrates metabolic and microbial cues into epithelial programs. Dysregulation of these pathways alters mucus output and glycan composition, lowers thresholds for immune tolerance, and links epithelial stress to chronic inflammation and neoplastic progression. Recognizing intracellular glycosylation as an actionable node, through quantitative readouts and pathway-targeted modulation, can refine glycan-informed diagnostics and enable mechanism-based interventions that complement strategies focused on luminal and terminal glycan structures. As this rapidly advancing area continues to resolve epithelial glycan–signal circuits with molecular precision, integrating intracellular and extracellular glycosylation layers will be essential for translating mucin biology into preventive and therapeutic benefit. Future research directions include elucidating the precise molecular mechanisms that regulate mucin glycosylation, developing advanced analytical techniques for glycomic analysis, and exploring glycosylation-based therapeutic interventions. These efforts will further clarify the role of mucin glycosylation in intestinal health and disease, and potentially yield novel therapeutic strategies for colonic disorders.

## Data Availability

All data generated or analyzed during this study are included in this published article.

## Abbreviations

The following abbreviations are used in this manuscript:

AB–PAS	Alcian Blue–Periodic Acid–Schiff
CD	Crohn’s disease
COSMC	Chaperone of C1GALT1 (C1GALT1C1)
CRC	Colorectal cancer
DC-SIGN	Dendritic cell-specific intercellular adhesion molecule-3-grabbing non-integrin
DMB	1,2-Diamino-4,5-methylenedioxybenzene
ER	Endoplasmic reticulum
FUT2	α1,2-Fucosyltransferase 2
GalNAc	N-Acetylgalactosamine
GALNT	Polypeptide N-acetylgalactosaminyltransferases
GCNT3	β1,6-N-acetylglucosaminyltransferase 3
GlcNAc	N-Acetylglucosamine
HID-AB	High iron diamine–Alcian Blue
IBD	Inflammatory bowel disease
IgA	Immunoglobulin A
LC-MS/MS	Liquid chromatography–tandem mass spectrometry
MALDI-TOF MS	Matrix-assisted laser desorption/ionization time-of-flight mass spectrometry
MGAT1	N-Acetylglucosaminyltransferase 1
MGAT2	N-Acetylglucosaminyltransferase 2
MGAT5	N-Acetylglucosaminyltransferase 5
MS	Mass spectrometry
MSI	Mass spectrometry imaging
Neu5Ac	N-Acetylneuraminic acid
O-GalNAc	O-linked N-acetylgalactosamine
O-GlcNAc	O-linked N-acetylglucosamine
OGT	O-GlcNAc transferase
OST	Oligosaccharyltransferase complex
PAPSS2	3′-Phosphoadenosine-5′-phosphosulfate synthase 2
PAS	Periodic Acid–Schiff
PNA	Peanut agglutinin
poly-LacNAc	Poly-N-acetyllactosamine
PUL	Polysaccharide utilization locus
SC	Secretory component
SIgA	Secretory immunoglobulin A
sTn	Sialyl-Tn antigen
Tn	Tn antigen
UC	Ulcerative colitis

## References

[1] Peterson L.W., Artis D. (2014). Intestinal epithelial cells: Regulators of barrier function and immune homeostasis. Nat. Rev. Immunol..

[2] Okumura R., Takeda K. (2018). Maintenance of intestinal homeostasis by mucosal barriers. Inflamm. Regen..

[3] Jäger S., Stange E.F., Wehkamp J. (2013). Inflammatory bowel disease: An impaired barrier disease. Langenbeck’s Arch. Surg..

[4] Chelakkot C., Ghim J., Ryu S.H. (2018). Mechanisms regulating intestinal barrier integrity and its pathological implications. Exp. Mol. Med..

[5] Dharmani P., Srivastava V., Kissoon-Singh V., Chadee K. (2009). Role of intestinal mucins in innate host defense mechanisms against pathogens. J. Innate Immun..

[6] McGuckin M.A., Lindén S.K., Sutton P., Florin T.H. (2011). Mucin dynamics and enteric pathogens. Nat. Rev. Microbiol..

[7] Grondin J.A., Kwon Y.H., Far P.M., Haq S., Khan W.I. (2020). Mucins in Intestinal Mucosal Defense and Inflammation: Learning from Clinical and Experimental Studies. Front. Immunol..

[8] Pelaseyed T., Bergström J.H., Gustafsson J.K., Ermund A., Birchenough G.M.H., Schütte A., van der Post S., Svensson F., Rodríguez-Piñeiro A.M., Nyström E.E.L. (2014). The mucus and mucins of the goblet cells and enterocytes provide the first defense line of the gastrointestinal tract and interact with the immune system. Immunol. Rev..

[9] Hansson G.C. (2020). Mucins and the Microbiome. Annu. Rev. Biochem..

[10] Linden S.K., Sutton P., Karlsson N.G., Korolik V., McGuckin M.A. (2008). Mucins in the mucosal barrier to infection. Mucosal Immunol..

[11] Petrosyan A., Ali M.F., Cheng P.-W. (2012). Glycosyltransferase-specific Golgi-targeting Mechanisms. J. Biol. Chem..

[12] Bergstrom K.S.B., Xia L. (2013). Mucin-type O-glycans and their roles in intestinal homeostasis. Glycobiology.

[13] Cheng H., Li H., Li Z., Wang Y., Liu L., Wang J., Ma X., Tan B. (2025). The role of glycosylated mucins in maintaining intestinal homeostasis and gut health. Anim. Nutr..

[14] Fekete E., Buret A.G. (2023). The role of mucin O-glycans in microbiota dysbiosis, intestinal homeostasis, and host-pathogen interactions. Am. J. Physiol.-Gastrointest. Liver Physiol..

[15] Kim Y.S., Ho S.B. (2010). Intestinal goblet cells and mucins in health and disease: Recent insights and progress. Curr. Gastroenterol. Rep..

[16] Brazil J.C., Parkos C.A. (2022). Finding the sweet spot: Glycosylation mediated regulation of intestinal inflammation. Mucosal Immunol..

[17] Robbe C., Capon C., Maes E., Rousset M., Zweibaum A., Zanetta J.-P., Michalski J.-C. (2003). Evidence of Regio-specific Glycosylation in Human Intestinal Mucins: Presence of an Acidic Gradient along the Intestinal Tract. J. Biol. Chem..

[18] Thomsson K.A., Holmén-Larsson J.M., Angström J., Johansson M.E., Xia L., Hansson G.C. (2012). Detailed O-glycomics of the Muc2 mucin from colon of wild-type, core 1- and core 3-transferase-deficient mice highlights differences compared with human MUC2. Glycobiology.

[19] He M., Zhou X., Wang X. (2024). Glycosylation: Mechanisms, biological functions and clinical implications. Signal Transduct. Target. Ther..

[20] Wandall H.H., Nielsen M.A.I., King-Smith S., de Haan N., Bagdonaite I. (2021). Global functions of O-glycosylation: Promises and challenges in O-glycobiology. FEBS J..

[21] Jin C., Kenny D.T., Skoog E.C., Padra M., Adamczyk B., Vitizeva V., Thorell A., Venkatakrishnan V., Lindén S.K., Karlsson N.G. (2017). Structural Diversity of Human Gastric Mucin Glycans. Mol. Cell. Proteom..

[22] Arike L., Hansson G.C. (2016). The Densely O-Glycosylated MUC2 Mucin Protects the Intestine and Provides Food for the Commensal Bacteria. J. Mol. Biol..

[23] Zhang Y., Sun L., Lei C., Li W., Han J., Zhang J., Zhang Y. (2022). A Sweet Warning: Mucin-Type O-Glycans in Cancer. Cells.

[24] Boottanun P., Ino Y., Shimada K., Hiraoka N., Angata K., Narimatsu H. (2021). Association between the expression of core 3 synthase and survival outcomes of patients with cholangiocarcinoma. Oncol. Lett..

[25] Gill D.J., Chia J., Senewiratne J., Bard F. (2010). Regulation of O-glycosylation through Golgi-to-ER relocation of initiation enzymes. J. Cell Biol..

[26] Parada Venegas D., De la Fuente M.K., Landskron G., González M.J., Quera R., Dijkstra G., Harmsen H.J.M., Faber K.N., Hermoso M.A. (2019). Short Chain Fatty Acids (SCFAs)-Mediated Gut Epithelial and Immune Regulation and Its Relevance for Inflammatory Bowel Diseases. Front. Immunol..

[27] Huang Z., Ding C., Huang X., Sun C., Zhong L. (2022). Exposure to 10 Hz Pulsed Magnetic Field Induced Slight Apoptosis and Reactive Oxygen Species in Primary Human Gingival Fibroblasts. Bioelectromagnetics.

[28] Maares M., Keil C., Straubing S., Robbe-Masselot C., Haase H. (2020). Zinc Deficiency Disturbs Mucin Expression, O-Glycosylation and Secretion by Intestinal Goblet Cells. Int. J. Mol. Sci..

[29] Hansson G.C. (2023). A Golgi oxygen sensor controls intestinal mucin glycosylation. EMBO J..

[30] de las Rivas M., Lira-Navarrete E., Gerken T.A., Hurtado-Guerrero R. (2019). Polypeptide GalNAc-Ts: From redundancy to specificity. Curr. Opin. Struct. Biol..

[31] Birchenough G.M.H., Johansson M.E., Gustafsson J.K., Bergström J.H., Hansson G.C. (2015). New developments in goblet cell mucus secretion and function. Mucosal Immunol..

[32] Fu J., Wei B., Wen T., Johansson M.E.V., Liu X., Bradford E., Thomsson K.A., McGee S., Mansour L., Tong M. (2011). Loss of intestinal core 1–derived O-glycans causes spontaneous colitis in mice. J. Clin. Investig..

[33] Bergstrom K., Fu J., Johansson M.E., Liu X., Gao N., Wu Q., Song J., McDaniel J.M., McGee S., Chen W. (2017). Core 1- and 3-derived O-glycans collectively maintain the colonic mucus barrier and protect against spontaneous colitis in mice. Mucosal Immunol..

[34] Elzinga J., Narimatsu Y., de Haan N., Clausen H., de Vos W.M., Tytgat H.L.P. (2024). Binding of *Akkermansia muciniphila* to mucin is O-glycan specific. Nat. Commun..

[35] Tailford L.E., Crost E.H., Kavanaugh D., Juge N. (2015). Mucin glycan foraging in the human gut microbiome. Front. Genet..

[36] An G., Wei B., Xia B., McDaniel J.M., Ju T., Cummings R.D., Braun J., Xia L. (2007). Increased susceptibility to colitis and colorectal tumors in mice lacking core 3-derived O-glycans. J. Exp. Med..

[37] Johansson M.E.V., Gustafsson J.K., Holmén-Larsson J., Jabbar K.S., Xia L., Xu H., Ghishan F.K., Carvalho F.A., Gewirtz A.T., Sjövall H. (2014). Bacteria penetrate the normally impenetrable inner colon mucus layer in both murine colitis models and patients with ulcerative colitis. Gut.

[38] Yang L., Li J., Lai J., Luan H., Cai Z., Wang Y., Zhao Y., Wu Y. (2016). Placental Transfer of Perfluoroalkyl Substances and Associations with Thyroid Hormones: Beijing Prenatal Exposure Study. Sci. Rep..

[39] Holst S., Wuhrer M., Rombouts Y., Drake R.R., Ball L.E. (2015). Chapter Six—Glycosylation Characteristics of Colorectal Cancer. Advances in Cancer Research.

[40] Bergstrom K., Liu X., Zhao Y., Gao N., Wu Q., Song K., Cui Y., Li Y., McDaniel J.M., McGee S. (2016). Defective Intestinal Mucin-Type O-Glycosylation Causes Spontaneous Colitis-Associated Cancer in Mice. Gastroenterology.

[41] González-Morelo K.J., Vega-Sagardía M., Garrido D. (2020). Molecular Insights Into O-Linked Glycan Utilization by Gut Microbes. Front. Microbiol..

[42] Wu X., Conlin V.S., Morampudi V., Ryz N.R., Nasser Y., Bhinder G., Bergstrom K.S., Yu H.B., Waterhouse C.C.M., Buchan A.M.J. (2015). Vasoactive Intestinal Polypeptide Promotes Intestinal Barrier Homeostasis and Protection Against Colitis in Mice. PLoS ONE.

[43] Jensen P.H., Kolarich D., Packer N.H. (2010). Mucin-type O-glycosylation—putting the pieces together. FEBS J..

[44] Pule M.A., Savoldo B., Myers G.D., Rossig C., Russell H.V., Dotti G., Huls M.H., Liu E., Gee A.P., Mei Z. (2008). Virus-specific T cells engineered to coexpress tumor-specific receptors: Persistence and antitumor activity in individuals with neuroblastoma. Nat. Med..

[45] Madunić K., Mayboroda O.A., Zhang T., Weber J., Boons G.J., Morreau H., van Vlierberghe R., van Wezel T., Lageveen-Kammeijer G.S.M., Wuhrer M. (2022). Specific (sialyl-)Lewis core 2 O-glycans differentiate colorectal cancer from healthy colon epithelium. Theranostics.

[46] Xiao S., Yang C., Zhang Y., Lai C. (2022). Downregulation of B3GNT6 is a predictor of poor outcomes in patients with colorectal cancer. World J. Surg. Oncol..

[47] Brockhausen I., Stanley P., Varki A., Cummings R.D., Esko J.D., Stanley P., Hart G.W., Aebi M., Darvill A.G., Kinoshita T., Packer N.H., Prestegard J.H. (2015). O-GalNAc Glycans. Essentials of Glycobiology.

[48] Kvorjak M., Ahmed Y., Miller M.L., Sriram R., Coronnello C., Hashash J.G., Hartman D.J., Telmer C.A., Miskov-Zivanov N., Finn O.J. (2020). Cross-talk between Colon Cells and Macrophages Increases ST6GALNAC1 and MUC1-sTn Expression in Ulcerative Colitis and Colitis-Associated Colon Cancer. Cancer Immunol. Res..

[49] Kraski A., Mousavi S., Heimesaat M.M., Bereswill S., Einspanier R., Alter T., Gölz G., Sharbati S. (2023). miR-125a-5p regulates the sialyltransferase ST3GAL1 in murine model of human intestinal campylobacteriosis. Gut Pathog..

[50] Pham T.A., Clare S., Goulding D., Arasteh J.M., Stares M.D., Browne H.P., Keane J.A., Page A.J., Kumasaka N., Kane L. (2014). Epithelial IL-22RA1-mediated fucosylation promotes intestinal colonization resistance to an opportunistic pathogen. Cell Host Microbe.

[51] Carroll D.J., Burns M.W.N., Mottram L., Propheter D.C., Boucher A., Lessen G.M., Kumar A., Malaker S.A., Xing C., Hooper L.V. (2022). Interleukin-22 regulates B3GNT7 expression to induce fucosylation of glycoproteins in intestinal epithelial cells. J. Biol. Chem..

[52] Raouf A.H., Tsai H.H., Parker N., Hoffman J., Walker R.J., Rhodes J.M. (1992). Sulphation of colonic and rectal mucin in inflammatory bowel disease: Reduced sulphation of rectal mucus in ulcerative colitis. Clin. Sci..

[53] Stanley P., Taniguchi N., Aebi M., Varki A., Cummings R.D., Esko J.D., Stanley P., Hart G.W., Aebi M., Darvill A.G., Kinoshita T., Packer N.H., Prestegard J.H. (2015). N-Glycans. Essentials of Glycobiology.

[54] Heazlewood C.K., Cook M.C., Eri R., Price G.R., Tauro S.B., Taupin D., Thornton D.J., Png C.W., Crockford T.L., Cornall R.J. (2008). Aberrant Mucin Assembly in Mice Causes Endoplasmic Reticulum Stress and Spontaneous Inflammation Resembling Ulcerative Colitis. PLOS Med..

[55] Ileri Z., Baka Z.M., Akin M., Apiliogullari S., Basciftci F.A. (2016). Effect of menstrual cycle on orthodontic pain perception: A controlled clinical trial. J. Orofac. Orthop..

[56] Asker N., Axelsson M.A.B., Olofsson S.-O., Hansson G.C. (1998). Dimerization of the human MUC2 mucin in the endoplasmic reticulum is followed by a N-glycosylation-dependent transfer of the mono- and dimers to the Golgi apparatus. J. Biol. Chem..

[57] Chugh S., Gnanapragassam V.S., Jain M., Rachagani S., Ponnusamy M.P., Batra S.K. (2015). Pathobiological implications of mucin glycans in cancer: Sweet poison and novel targets. Biochim. Biophys. Acta.

[58] Beaman E.-M., Carter D.R.F., Brooks S.A. (2022). GALNTs: Master regulators of metastasis-associated epithelial-mesenchymal transition (EMT)?. Glycobiology.

[59] McCool D.J., Okada Y., Forstner J.F., Forstner G.G. (1999). Roles of calreticulin and calnexin during mucin synthesis in LS180 and HT29/A1 human colonic adenocarcinoma cells. Biochem. J..

[60] Paone P., Cani P.D. (2020). Mucus barrier, mucins and gut microbiota: The expected slimy partners?. Gut.

[61] Hasnain S.Z., Tauro S., Das I., Tong H., Chen A.C.H., Jeffery P.L., McDonald V., Florin T.H., McGuckin M.A. (2013). IL-10 Promotes Production of Intestinal Mucus by Suppressing Protein Misfolding and Endoplasmic Reticulum Stress in Goblet Cells. Gastroenterology.

[62] Javitt G., Khmelnitsky L., Albert L., Bigman L.S., Elad N., Morgenstern D., Ilani T., Levy Y., Diskin R., Fass D. (2020). Assembly Mechanism of Mucin and von Willebrand Factor Polymers. Cell.

[63] Bäckström M., Ambort D., Thomsson E., Johansson M.E., Hansson G.C. (2013). Increased understanding of the biochemistry and biosynthesis of MUC2 and other gel-forming mucins through the recombinant expression of their protein domains. Mol. Biotechnol..

[64] Roy A., Meregini S., Cho H.J., Chen Z., Zaki A., Argula T., Beutler B., SoRelle J.A. (2025). N-glycosylation enzyme Mpi is essential for mucin O-glycosylation, host-microbe homeostasis, Paneth cell defense, and metabolism. Res. Sq..

[65] Sun L., Zhang Y., Li W., Zhang J., Zhang Y. (2023). Mucin Glycans: A Target for Cancer Therapy. Molecules.

[66] Axelsson M.A.B., Karlsson N.G., Steel D.M., Ouwendijk J., Nilsson T., Hansson G.C. (2001). Neutralization of pH in the Golgi apparatus causes redistribution of glycosyltransferases and changes in the O-glycosylation of mucins. Glycobiology.

[67] Steentoft C., Vakhrushev S.Y., Joshi H.J., Kong Y., Vester-Christensen M.B., Schjoldager K.T.B.G., Lavrsen K., Dabelsteen S., Pedersen N.B., Marcos-Silva L. (2013). Precision mapping of the human O-GalNAc glycoproteome through SimpleCell technology. EMBO J..

[68] Chandrasekaran E.V., Xue J., Xia J., Locke R.D., Patil S.A., Neelamegham S., Matta K.L. (2011). Mammalian Sialyltransferase ST3Gal-II: Its Exchange Sialylation Catalytic Properties Allow Labeling of Sialyl Residues in Mucin-Type Sialylated Glycoproteins and Specific Gangliosides. Biochemistry.

[69] Venturi G., Gomes Ferreira I., Pucci M., Ferracin M., Malagolini N., Chiricolo M., Dall’Olio F. (2019). Impact of sialyltransferase ST6GAL1 overexpression on different colon cancer cell types. Glycobiology.

[70] Yao Y., Kim G., Shafer S., Chen Z., Kubo S., Ji Y., Luo J., Yang W., Perner S.P., Kanellopoulou C. (2022). Mucus sialylation determines intestinal host-commensal homeostasis. Cell.

[71] Taniguchi M., Okumura R., Matsuzaki T., Nakatani A., Sakaki K., Okamoto S., Ishibashi A., Tani H., Horikiri M., Kobayashi N. (2023). Sialylation shapes mucus architecture inhibiting bacterial invasion in the colon. Mucosal Immunol..

[72] Martens E.C., Neumann M., Desai M.S. (2018). Interactions of commensal and pathogenic microorganisms with the intestinal mucosal barrier. Nat. Rev. Microbiol..

[73] Goto Y., Obata T., Kunisawa J., Sato S., Ivanov I.I., Lamichhane A., Takeyama N., Kamioka M., Sakamoto M., Matsuki T. (2014). Innate lymphoid cells regulate intestinal epithelial cell glycosylation. Science.

[74] Koropatkin N.M., Cameron E.A., Martens E.C. (2012). How glycan metabolism shapes the human gut microbiota. Nat. Rev. Microbiol..

[75] Zhang N.Z., Zhao L.F., Zhang Q., Fang H., Song W.L., Li W.Z., Ge Y.S., Gao P. (2023). Core fucosylation and its roles in gastrointestinal glycoimmunology. World J. Gastrointest. Oncol..

[76] Pinho S.S., Reis C.A. (2015). Glycosylation in cancer: Mechanisms and clinical implications. Nat. Rev. Cancer.

[77] van der Post S., Subramani D.B., Bäckström M., Johansson M.E.V., Vester-Christensen M.B., Mandel U., Bennett E.P., Clausen H., Dahlén G., Sroka A. (2013). Site-specific O-Glycosylation on the MUC2 Mucin Protein Inhibits Cleavage by the *Porphyromonas gingivalis* Secreted Cysteine Protease (RgpB). J. Biol. Chem..

[78] Keir M., Yi Y., Lu T., Ghilardi N. (2020). The role of IL-22 in intestinal health and disease. J. Exp. Med..

[79] Moriwaki K., Miyoshi E. (2010). Fucosylation and gastrointestinal cancer. World J. Hepatol..

[80] Tobisawa Y., Imai Y., Fukuda M., Kawashima H. (2010). Sulfation of colonic mucins by N-acetylglucosamine 6-O-sulfotransferase-2 and its protective function in experimental colitis in mice. J. Biol. Chem..

[81] Rosen S.D., Taniguchi N., Honke K., Fukuda M., Narimatsu H., Yamaguchi Y., Angata T. (2014). Carbohydrate (N-Acetylglucosamine 6-O) Sulfotransferase 4 (CHST4). Handbook of Glycosyltransferases and Related Genes.

[82] Luis A.S., Hansson G.C. (2023). Intestinal mucus and their glycans: A habitat for thriving microbiota. Cell Host Microbe.

[83] Lennon G., Balfe Á., Bambury N., Lavelle A., Maguire A., Docherty N.G., Coffey J.C., Winter D.C., Sheahan K., O’Connell P.R. (2014). Correlations between colonic crypt mucin chemotype, inflammatory grade and Desulfovibrio species in ulcerative colitis. Color. Dis..

[84] Meldrum O.W., Yakubov G.E., Bonilla M.R., Deshmukh O., McGuckin M.A., Gidley M.J. (2018). Mucin gel assembly is controlled by a collective action of non-mucin proteins, disulfide bridges, Ca^2+^-mediated links, and hydrogen bonding. Sci. Rep..

[85] Hasnain S.Z., Dawson P.A., Lourie R., Hutson P., Tong H., Grencis R.K., McGuckin M.A., Thornton D.J. (2017). Immune-driven alterations in mucin sulphation is an important mediator of Trichuris muris helminth expulsion. PLoS Pathog..

[86] Johansson M.E., Larsson J.M., Hansson G.C. (2011). The two mucus layers of colon are organized by the MUC2 mucin, whereas the outer layer is a legislator of host-microbial interactions. Proc. Natl. Acad. Sci. USA.

[87] Tokuhara D., Kurashima Y., Kamioka M., Nakayama T., Ernst P., Kiyono H. (2019). A comprehensive understanding of the gut mucosal immune system in allergic inflammation. Allergol. Int..

[88] Zheng D., Liwinski T., Elinav E. (2020). Interaction between microbiota and immunity in health and disease. Cell Res..

[89] Johansson M.E.V., Jakobsson H.E., Holmén-Larsson J., Schütte A., Ermund A., Rodríguez-Piñeiro A.M., Arike L., Wising C., Svensson F., Bäckhed F. (2015). Normalization of Host Intestinal Mucus Layers Requires Long-Term Microbial Colonization. Cell Host Microbe.

[90] Hansson G.C. (2012). Role of mucus layers in gut infection and inflammation. Curr. Opin. Microbiol..

[91] Ambort D., Johansson M.E.V., Gustafsson J.K., Nilsson H.E., Ermund A., Johansson B.R., Koeck P.J.B., Hebert H., Hansson G.C. (2012). Calcium and pH-dependent packing and release of the gel-forming MUC2 mucin. Proc. Natl. Acad. Sci. USA.

[92] Stanforth K.J., Zakhour M.I., Chater P.I., Wilcox M.D., Adamson B., Robson N.A., Pearson J.P. (2024). The MUC2 Gene Product: Polymerisation and Post-Secretory Organisation—Current Models. Polymers.

[93] Gustafsson J.K., Ermund A., Johansson M.E.V., Schütte A., Hansson G.C., Sjövall H. (2012). An ex vivo method for studying mucus formation, properties, and thickness in human colonic biopsies and mouse small and large intestinal explants. Am. J. Physiol.-Gastrointest. Liver Physiol..

[94] Johansson M.E.V., Phillipson M., Petersson J., Velcich A., Holm L., Hansson G.C. (2008). The inner of the two Muc2 mucin-dependent mucus layers in colon is devoid of bacteria. Proc. Natl. Acad. Sci. USA.

[95] Johansson M.E.V., Ambort D., Pelaseyed T., Schütte A., Gustafsson J.K., Ermund A., Subramani D.B., Holmén-Larsson J.M., Thomsson K.A., Bergström J.H. (2011). Composition and functional role of the mucus layers in the intestine. Cell. Mol. Life Sci..

[96] Crouzier T., Boettcher K., Geonnotti A.R., Kavanaugh N.L., Hirsch J.B., Ribbeck K., Lieleg O. (2015). Modulating Mucin Hydration and Lubrication by Deglycosylation and Polyethylene Glycol Binding. Adv. Mater. Interfaces.

[97] Xu P., Xi Y., Zhu J., Zhang M., Luka Z., Stolz D.B., Cai X., Xie Y., Xu M., Ren S. (2021). Intestinal Sulfation Is Essential to Protect Against Colitis and Colonic Carcinogenesis. Gastroenterology.

[98] Yang S., Duncan G.A. (2023). Synthetic mucus biomaterials for antimicrobial peptide delivery. J. Biomed. Mater. Res. Part A.

[99] Johansson M.E., Hansson G.C. (2022). Goblet cells need some stress. J. Clin. Investig..

[100] Antonini M., Lo Conte M., Sorini C., Falcone M. (2019). How the Interplay Between the Commensal Microbiota, Gut Barrier Integrity, and Mucosal Immunity Regulates Brain Autoimmunity. Front. Immunol..

[101] Dias A.M., Pereira M.S., Padrão N.A., Alves I., Marcos-Pinto R., Lago P., Pinho S.S. (2018). Glycans as critical regulators of gut immunity in homeostasis and disease. Cell. Immunol..

[102] Pinho S.S., Alves I., Gaifem J., Rabinovich G.A. (2023). Immune regulatory networks coordinated by glycans and glycan-binding proteins in autoimmunity and infection. Cell. Mol. Immunol..

[103] Crespo H.J., Lau J.T., Videira P.A. (2013). Dendritic Cells: A Spot on Sialic Acid. Front. Immunol..

[104] Phalipon A., Cardona A., Kraehenbuhl J.P., Edelman L., Sansonetti P.J., Corthésy B. (2002). Secretory component: A new role in secretory IgA-mediated immune exclusion in vivo. Immunity.

[105] Gustafsson J.K., Davis J.E., Rappai T., McDonald K.G., Kulkarni D.H., Knoop K.A., Hogan S.P., Fitzpatrick J.A.J., Lencer W.I., Newberry R.D. (2021). Intestinal goblet cells sample and deliver lumenal antigens by regulated endocytic uptake and transcytosis. eLife.

[106] Wang B.X., Wheeler K.M., Cady K.C., Lehoux S., Cummings R.D., Laub M.T., Ribbeck K. (2021). Mucin Glycans Signal through the Sensor Kinase RetS to Inhibit Virulence-Associated Traits in Pseudomonas aeruginosa. Curr. Biol..

[107] Macauley M.S., Crocker P.R., Paulson J.C. (2014). Siglec-mediated regulation of immune cell function in disease. Nat. Rev. Immunol..

[108] Baggioli A., Famulari A. (2014). On the inter-ring torsion potential of regioregular P3HT: A first principles reexamination with explicit side chains. Phys. Chem. Chem. Phys..

[109] Leclaire C., Lecointe K., Gunning P.A., Tribolo S., Kavanaugh D.W., Wittmann A., Latousakis D., MacKenzie D.A., Kawasaki N., Juge N. (2018). Molecular basis for intestinal mucin recognition by galectin-3 and C-type lectins. FASEB J..

[110] Yu X., Qian J., Ding L., Yin S., Zhou L., Zheng S. (2023). Galectin-1: A Traditionally Immunosuppressive Protein Displays Context-Dependent Capacities. Int. J. Mol. Sci..

[111] Fernandez-Perez R., Lopez-Santalla M., Sánchez-Domínguez R., Alberquilla O., Gutiérrez-Cañas I., Juarranz Y., Bueren J.A., Garin M.I. (2021). Enhanced Susceptibility of Galectin-1 Deficient Mice to Experimental Colitis. Front. Immunol..

[112] Shen H., Lu Z., Xu Z., Shen Z. (2017). Diet-induced reconstruction of mucosal microbiota associated with alterations of epithelium lectin expression and regulation in the maintenance of rumen homeostasis. Sci. Rep..

[113] Zhao M., Xiong X., Ren K., Xu B., Cheng M., Sahu C., Wu K., Nie Y., Huang Z., Blumberg R.S. (2018). Deficiency in intestinal epithelial O-GlcNAcylation predisposes to gut inflammation. EMBO Mol. Med..

[114] Yang Y.R., Kim D.H., Seo Y.K., Park D., Jang H.J., Choi S.Y., Lee Y.H., Lee G.H., Nakajima K., Taniguchi N. (2015). Elevated O-GlcNAcylation promotes colonic inflammation and tumorigenesis by modulating NF-κB signaling. Oncotarget.

[115] Zhao M., Ren K., Xiong X., Xin Y., Zou Y., Maynard J.C., Kim A., Battist A.P., Koneripalli N., Wang Y. (2022). Epithelial STAT6 O-GlcNAcylation drives a concerted anti-helminth alarmin response dependent on tuft cell hyperplasia and Gasdermin C. Immunity.

[116] Desai M.S., Seekatz A.M., Koropatkin N.M., Kamada N., Hickey C.A., Wolter M., Pudlo N.A., Kitamoto S., Terrapon N., Muller A. (2016). A Dietary Fiber-Deprived Gut Microbiota Degrades the Colonic Mucus Barrier and Enhances Pathogen Susceptibility. Cell.

[117] Alves I., Fernandes Â., Santos-Pereira B., Azevedo C.M., Pinho S.S. (2022). Glycans as a key factor in self and nonself discrimination: Impact on the breach of immune tolerance. FEBS Lett..

[118] Lübbers J., Rodríguez E., van Kooyk Y. (2018). Modulation of Immune Tolerance via Siglec-Sialic Acid Interactions. Front. Immunol..

[119] Pederson K., Mitchell D.A., Prestegard J.H. (2014). Structural characterization of the DC-SIGN-Lewis^X^ complex. Biochemistry.

[120] Shan M., Gentile M., Yeiser J.R., Walland A.C., Bornstein V.U., Chen K., He B., Cassis L., Bigas A., Cols M. (2013). Mucus enhances gut homeostasis and oral tolerance by delivering immunoregulatory signals. Science.

[121] Bergstrom K., Xia L. (2022). The barrier and beyond: Roles of intestinal mucus and mucin-type O-glycosylation in resistance and tolerance defense strategies guiding host-microbe symbiosis. Gut Microbes.

[122] Juge N. (2019). Special Issue: Gut Bacteria-Mucus Interaction. Microorganisms.

[123] Li H., Limenitakis J.P., Fuhrer T., Geuking M.B., Lawson M.A., Wyss M., Brugiroux S., Keller I., Macpherson J.A., Rupp S. (2015). The outer mucus layer hosts a distinct intestinal microbial niche. Nat. Commun..

[124] Pickard J.M., Maurice C.F., Kinnebrew M.A., Abt M.C., Schenten D., Golovkina T.V., Bogatyrev S.R., Ismagilov R.F., Pamer E.G., Turnbaugh P.J. (2014). Rapid fucosylation of intestinal epithelium sustains host–commensal symbiosis in sickness. Nature.

[125] Jakobsson H.E., Rodríguez-Piñeiro A.M., Schütte A., Ermund A., Boysen P., Bemark M., Sommer F., Bäckhed F., Hansson G.C., Johansson M.E.V. (2015). The composition of the gut microbiota shapes the colon mucus barrier. EMBO Rep..

[126] McGovern D.P.B., Jones M.R., Taylor K.D., Marciante K., Yan X., Dubinsky M., Ippoliti A., Vasiliauskas E., Berel D., Derkowski C. (2010). Fucosyltransferase 2 (FUT2) non-secretor status is associated with Crohn’s disease. Hum. Mol. Genet..

[127] Arike L., Holmén-Larsson J., Hansson G.C. (2017). Intestinal Muc2 mucin O-glycosylation is affected by microbiota and regulated by differential expression of glycosyltranferases. Glycobiology.

[128] Sonnenburg E.D., Smits S.A., Tikhonov M., Higginbottom S.K., Wingreen N.S., Sonnenburg J.L. (2016). Diet-induced extinctions in the gut microbiota compound over generations. Nature.

[129] Shuoker B., Pichler M.J., Jin C., Sakanaka H., Wu H., Gascueña A.M., Liu J., Nielsen T.S., Holgersson J., Nordberg Karlsson E. (2023). Sialidases and fucosidases of *Akkermansia muciniphila* are crucial for growth on mucin and nutrient sharing with mucus-associated gut bacteria. Nat. Commun..

[130] Gaudier E., Forestier L., Gouyer V., Huet G., Julien R., Hoebler C. (2004). Butyrate regulation of glycosylation-related gene expression: Evidence for galectin-1 upregulation in human intestinal epithelial goblet cells. Biochem. Biophys. Res. Commun..

[131] Bresalier R.S., Ho S.B., Schoeppner H.L., Kim Y.S., Sleisenger M.H., Brodt P., Byrd J.C. (1996). Enhanced sialylation of mucin-associated carbohydrate structures in human colon cancer metastasis. Gastroenterology.

[132] de Oliveira R.M., Ornelas Ricart C.A., Araujo Martins A.M. (2018). Use of Mass Spectrometry to Screen Glycan Early Markers in Hepatocellular Carcinoma. Front. Oncol..

[133] Blaschke C.R.K., McDowell C.T., Black A.P., Mehta A.S., Angel P.M., Drake R.R. (2021). Glycan Imaging Mass Spectrometry: Progress in Developing Clinical Diagnostic Assays for Tissues, Biofluids, and Cells. Clin. Lab. Med..

[134] Mahoney K.E., Malaker S.A. (2024). Analysis of Mucin-Domain Glycoproteins Using Mass Spectrometry. Curr. Protoc..

[135] Corfield A. (2017). Eukaryotic protein glycosylation: A primer for histochemists and cell biologists. Histochem. Cell Biol..

[136] Vadivazhagan K., Amitkumar K., Sudalaimuthu M. (2022). Quantitative Analysis of Mucin Expression Using Combined Alcian Blue-Periodic Acid Schiff (AB-PAS) Stain and Combined High Iron Diamine-Alcian Blue (HID-AB) Stain and the Correlation with Histomorphological Score in Chronic Calculous Cholecystitis. Cureus.

[137] Kannan S., Lakku R.A., Niranjali D., Jayakumar K., Steven A.H., Taralakshmi V.V., Chandramohan S., Balakrishnan R., Schmidt C., Halagowder D. (2003). Expression of peanut agglutinin-binding mucin-type glycoprotein in human esophageal squamous cell carcinoma as a marker. Mol. Cancer.

[138] Domino S.E., Karnak D.M., Hurd E.A. (2007). Cell surface fucosylation does not affect development of colon tumors in mice with germline Smad3 mutation. Tumor Biol..

[139] Coletto E., Savva G.M., Latousakis D., Pontifex M., Crost E.H., Vaux L., Telatin A., Bergstrom K., Vauzour D., Juge N. (2023). Role of mucin glycosylation in the gut microbiota-brain axis of core 3 O-glycan deficient mice. Sci. Rep..

[140] Drake R.R., Powers T.W., Jones E.E., Bruner E., Mehta A.S., Angel P.M. (2017). MALDI Mass Spectrometry Imaging of N-Linked Glycans in Cancer Tissues. Adv. Cancer Res..

[141] Han S.-S., Shim H.-E., Park S.-J., Kim B.-C., Lee D.-E., Chung H.-M., Moon S.-H., Kang S.-W. (2018). Safety and Optimization of Metabolic Labeling of Endothelial Progenitor Cells for Tracking. Sci. Rep..

[142] Dube D.H., Prescher J.A., Quang C.N., Bertozzi C.R. (2006). Probing mucin-type O-linked glycosylation in living animals. Proc. Natl. Acad. Sci. USA.

[143] Hang H.C., Yu C., Kato D.L., Bertozzi C.R. (2003). A metabolic labeling approach toward proteomic analysis of mucin-type O-linked glycosylation. Proc. Natl. Acad. Sci. USA.

[144] Pedram K., Shon D.J., Tender G.S., Mantuano N.R., Northey J.J., Metcalf K.J., Wisnovsky S.P., Riley N.M., Forcina G.C., Malaker S.A. (2024). Design of a mucin-selective protease for targeted degradation of cancer-associated mucins. Nat. Biotechnol..

[145] Yu A.C.Y., Worrall L.J., Strynadka N.C.J. (2012). Structural Insight into the Bacterial Mucinase StcE Essential to Adhesion and Immune Evasion during Enterohemorrhagic *E. coli* Infection. Structure.

[146] Schumacher U., Mitchell B.S. (1998). Use of fluorochrome-labeled lectins in light microscopy. Methods Mol. Med..

[147] Nason R., Büll C., Konstantinidi A., Sun L., Ye Z., Halim A., Du W., Sørensen D.M., Durbesson F., Furukawa S. (2021). Display of the human mucinome with defined O-glycans by gene engineered cells. Nat. Commun..

[148] Xie Y., Sheng Y., Li Q., Ju S., Reyes J., Lebrilla C.B. (2020). Determination of the glycoprotein specificity of lectins on cell membranes through oxidative proteomics. Chem. Sci..

[149] He B.J., Liu Z.K., Shen P., Sun Y.X., Chen B., Zhan S.Y., Lin H.B. (2022). Epidemiological study on the incidence of inflammatory bowel disease in Yinzhou District, Ningbo City from 2011 to 2020. J. Peking Univ. Health Sci..

[150] Alatab S., Sepanlou S.G., Ikuta K., Vahedi H., Bisignano C., Safiri S., Sadeghi A., Nixon M.R., Abdoli A., Abolhassani H. (2020). The global, regional, and national burden of inflammatory bowel disease in 195 countries and territories, 1990–2017: A systematic analysis for the Global Burden of Disease Study 2017. Lancet Gastroenterol. Hepatol..

[151] Larsson J.M., Karlsson H., Crespo J.G., Johansson M.E., Eklund L., Sjövall H., Hansson G.C. (2011). Altered O-glycosylation profile of MUC2 mucin occurs in active ulcerative colitis and is associated with increased inflammation. Inflamm. Bowel Dis..

[152] Tsai H.H., Hart C.A., Rhodes J.M. (1991). Production of mucin degrading sulphatase and glycosidases by Bacteroides thetaiotaomicron. Lett. Appl. Microbiol..

[153] Abo H., Muraki A., Harusato A., Imura T., Suzuki M., Takahashi K., Denning T.L., Kawashima H. (2023). N-acetylglucosamine-6-O-sulfation on intestinal mucins prevents obesity and intestinal inflammation by regulating gut microbiota. JCI Insight.

[154] Xia B., Zhong R., Wu W., Luo C., Meng Q., Gao Q., Zhao Y., Chen L., Zhang S., Zhao X. (2022). Mucin O-glycan-microbiota axis orchestrates gut homeostasis in a diarrheal pig model. Microbiome.

[155] Fang J., Wang H., Zhou Y., Zhang H., Zhou H., Zhang X. (2021). Slimy partners: The mucus barrier and gut microbiome in ulcerative colitis. Exp. Mol. Med..

[156] Feofanova N.A., Bets V.D., Borisova M.A., Litvinova E.A. (2022). L-fucose reduces gut inflammation due to T-regulatory response in Muc2 null mice. PLoS ONE.

[157] Dong K., Deng S.J., He B.Y., Guo Z.Y., Guan Z.L., Leng X., Ma R.R., Wang D.Y., Xing J.F., You C.Y. (2023). Mucoadhesive Nanoparticles Enhance the Therapeutic Effect of Dexamethasone on Experimental Ulcerative Colitis by the Local Administration as an Enema. Drug Des. Dev. Ther..

[158] Moulari B., Béduneau A., Pellequer Y., Lamprecht A. (2014). Lectin-decorated nanoparticles enhance binding to the inflamed tissue in experimental colitis. J. Control. Release.

[159] Chandrasekaran P., Weiskirchen S., Weiskirchen R. (2024). Effects of Probiotics on Gut Microbiota: An Overview. Int. J. Mol. Sci..

[160] Caballero-Franco C., Keller K., De Simone C., Chadee K. (2007). The VSL#3 probiotic formula induces mucin gene expression and secretion in colonic epithelial cells. Am. J. Physiol.-Gastrointest. Liver Physiol..

[161] Huang C., Hao W., Wang X., Zhou R., Lin Q. (2023). Probiotics for the treatment of ulcerative colitis: A review of experimental research from 2018 to 2022. Front. Microbiol..

[162] Huang Y.L., Chassard C., Hausmann M., von Itzstein M., Hennet T. (2015). Sialic acid catabolism drives intestinal inflammation and microbial dysbiosis in mice. Nat. Commun..

[163] Kang Y., Park H., Choe B.-H., Kang B. (2022). The Role and Function of Mucins and Its Relationship to Inflammatory Bowel Disease. Front. Med..

[164] Corfield A.P., Myerscough N., Bradfield N., Corfield Cdo A., Gough M., Clamp J.R., Durdey P., Warren B.F., Bartolo D.C., King K.R. (1996). Colonic mucins in ulcerative colitis: Evidence for loss of sulfation. Glycoconj. J..

[165] Hashash J.G., Beatty P.L., Critelli K., Hartman D.J., Regueiro M., Tamim H., Regueiro M.D., Binion D.G., Finn O.J. (2021). Altered Expression of the Epithelial Mucin MUC1 Accompanies Endoscopic Recurrence of Postoperative Crohn’s Disease. J. Clin. Gastroenterol..

[166] Robbe Masselot C., Cordier C., Marsac B., Nachury M., Léonard R., Sendid B. (2023). Human Fecal Mucin Glycosylation as a New Biomarker in Inflammatory Bowel Diseases. Inflamm. Bowel Dis..

[167] Salvatore S., Heuschkel R., Tomlin S., Davies S., Edwards S., Walker-Smith J., French I., Murch S. (2000). A pilot study of N-acetyl glucosamine, a nutritional substrate for glycosaminoglycan synthesis, in paediatric chronic inflammatory bowel disease. Aliment. Pharmacol. Ther..

[168] Dias A.M., Correia A., Pereira M.S., Almeida C.R., Alves I., Pinto V., Catarino T.A., Mendes N., Leander M., Oliva-Teles M.T. (2018). Metabolic control of T cell immune response through glycans in inflammatory bowel disease. Proc. Natl. Acad. Sci. USA.

[169] Schroeder B.O., Birchenough G.M.H., Ståhlman M., Arike L., Johansson M.E.V., Hansson G.C., Bäckhed F. (2018). Bifidobacteria or Fiber Protects against Diet-Induced Microbiota-Mediated Colonic Mucus Deterioration. Cell Host Microbe.

[170] Xia L. (2010). Core 3-derived O-glycans are essential for intestinal mucus barrier function. Methods Enzymol..

[171] Guo M., Xing D., Wang J., Zhang Y., Li Z., Jiao X. (2023). Potent Intestinal Mucosal Barrier Enhancement of Nostoc commune Vaucher Polysaccharide Supplementation Ameliorates Acute Ulcerative Colitis in Mice Mediated by Gut Microbiota. Nutrients.

[172] Sun Q.H., Wang Y.S., Liu G., Zhou H.L., Jian Y.P., Liu M.D., Zhang D., Ding Q., Zhao R.X., Chen J.F. (2020). Enhanced O-linked Glcnacylation in Crohn’s disease promotes intestinal inflammation. eBioMedicine.

[173] Bu X.D., Li N., Tian X.Q., Li L., Wang J.S., Yu X.J., Huang P.L. (2010). Altered expression of MUC2 and MUC5AC in progression of colorectal carcinoma. World J. Gastroenterol..

[174] Wu M., Wu Y., Li J., Bao Y., Guo Y., Yang W. (2018). The Dynamic Changes of Gut Microbiota in Muc2 Deficient Mice. Int. J. Mol. Sci..

[175] Beatson R., Tajadura-Ortega V., Achkova D., Picco G., Tsourouktsoglou T.-D., Klausing S., Hillier M., Maher J., Noll T., Crocker P.R. (2016). The mucin MUC1 modulates the tumor immunological microenvironment through engagement of the lectin Siglec-9. Nat. Immunol..

[176] Hung J.-S., Huang J., Lin Y.-C., Huang M.-J., Lee P.-H., Lai H.-S., Liang J.-T., Huang M.-C. (2014). C1GALT1 overexpression promotes the invasive behavior of colon cancer cells through modifying O-glycosylation of FGFR2. Oncotarget.

[177] Chien M.-W., Fu S.-H., Hsu C.-Y., Liu Y.-W., Sytwu H.-K. (2018). The Modulatory Roles of N-glycans in T-Cell-Mediated Autoimmune Diseases. Int. J. Mol. Sci..

[178] Habeeb I.F., Alao T.E., Delgado D., Buffone A. (2024). When a negative (charge) is not a positive: Sialylation and its role in cancer mechanics and progression. Front. Oncol..

[179] Pienkowski T., Wawrzak-Pienkowska K., Tankiewicz-Kwedlo A., Ciborowski M., Kurek K., Pawlak D. (2025). Leveraging glycosylation for early detection and therapeutic target discovery in pancreatic cancer. Cell Death Dis..

[180] Yang Y., Yin Y., Xu W., Kang Y., Chen J., Zou Y., Xiao Z., Li Z., Cao P. (2024). Restoring O-glycosylation and expression of MUC2 limits progression of colorectal cancer. bioRxiv.

[181] Adis Editorial (2003). Cancer vaccine THERATOPE- Biomira. Drugs R D.

[182] Berois N., Pittini A., Osinaga E. (2022). Targeting Tumor Glycans for Cancer Therapy: Successes, Limitations, and Perspectives. Cancers.

[183] Cornelissen L.A.M., Blanas A., Zaal A., van der Horst J.C., Kruijssen L.J.W., O’Toole T., van Kooyk Y., van Vliet S.J. (2020). Tn Antigen Expression Contributes to an Immune Suppressive Microenvironment and Drives Tumor Growth in Colorectal Cancer. Front. Oncol..

[184] Do K.T., Chow L.Q.M., Reckamp K., Sanborn R.E., Burris H., Robert F., Camidge D.R., Steuer C.E., Strickler J.H., Weise A. (2021). First-In-Human, First-In-Class, Phase I Trial of the Fucosylation Inhibitor SGN-2FF in Patients with Advanced Solid Tumors. Oncologist.

[185] Wang M., Zhang Z., Chen M., Lv Y., Tian S., Meng F., Zhang Y., Guo X., Chen Y., Yang M. (2023). FDW028, a novel FUT8 inhibitor, impels lysosomal proteolysis of B7-H3 via chaperone-mediated autophagy pathway and exhibits potent efficacy against metastatic colorectal cancer. Cell Death Dis..

